# Supplementary material for: Exploratory randomised trial of face-to-face and mobile phone counselling against usual care for tobacco cessation in Indian primary care: a randomised controlled trial protocol for project CERTAIN
Source: BMJ Open. 2022 Jan 6;12(1):e048628. doi: 10.1136/bmjopen-2021-048628 (PMC8739426; doi:10.1136/bmjopen-2021-048628)
Supplement: Supplementary data [file bmjopen-2021-048628supp001.pdf]

BMJ Publishing Group Limited (BMJ) disclaims all liability and responsibility arising from any reliance placed on this supplemental material which has been supplied by the author(s)

Appendix A: Details about the Formative Research phase of CERTAIN Study

Qualitative research methods will be used in the formative phase. In-Depth Interviews (IDIs) will be conducted with smokeless tobacco (SLT) users as well as primary care physicians present in UPHCs and Focus Group Discussions (FGDs) will be conducted with tobacco cessation counsellors. The formative research study will be based on a maximum variation sampling. A maximum variation sample will be constructed by identifying key dimensions of variations and then finding participants that vary from each other as much as possible. This sampling will yield detailed descriptions of each respondent, useful for documenting uniqueness, and this will allow researchers to identify common themes that are evident across the heterogeneous group of study participants. The variation will cover the socio-demographic characteristics of the study participants. The formative phase will assist in finalising two components of the intervention, contents for face to face counselling and a mobile phone intervention (regular messages). The formative research will be conducted in three sequential phases.

Phases in Formative Research:

In Phase 1, IDIs will be conducted with a purposive sample of male (n=15) and female (n=15) SLT users or dual users visiting UPHCs during the study period. IDIs will also be conducted with primary care physicians (n=5) present at these UPHCs. Participants' views on the detail of text messages will be explored. These views will inform the final text messages deemed suitable for tobacco cessation support. The messages will be tailored to users' health conditions and will use local terms, dialects and opinions of various stakeholders.

In Phase 2A, two FGDs will be conducted each with six primary care staff (ANM, ASHA, pharmacists, staff nurse, counsellors) who will be employed in the urban health clinic for more than a year and who show willingness to participate. Focus groups will explore the perspective of participants' regarding the preliminary content and structure of messages, use of tailored messages addressing the risk of tobacco for specific diseases, associated comorbidities, risks of tobacco usage in these comorbid conditions, readiness to quit stage and withdrawal symptoms.

In Phase 2B, developed text messages will undergo validation. The validation study will be conducted with SLT users visiting UPHCs. Each will be given a validation tool (developed specifically for this study) which will be used to score each of the newly developed messages. The messages that get the highest score will be adopted as a part of the intervention. IDIs will also be conducted with these tobacco users to gain their views and opinions regarding developed text messages.

Phase 3, will be conducted at two-time points (i.e., mid-line and end-line) in the trial follow-up period. At mid-line (1.5 months), IDIs will be conducted with SLT users to assess the appropriate engagement of the text messages. At end of three-months, IDIs will be conducted with SLT users who complete the follow-up period as well as with the SLT users who drop out of the study. Perceptions about appropriate engagement and satisfaction with text messages will be captured at this point.

The IDIs and FGDs will be conducted by trained qualitative research team members. Each team will have a moderator and note-taker. All interviews will be audio-recorded (with participants' consent) and transcribed. The transcriptions will be analysed using a qualitative framework analysis method. All transcripts will be reviewed and coded by two members of the research team to minimize subjective interpretations. We will use Atlas.ti (Version 8) software to evaluate the data.

Training of qualitative research team: All members of the qualitative research team participating in the study at each UPHCs will be trained. The training of qualitative researchers will include how to introduce the study to potential participants, obtaining verbal and written consent, conducting IDIs and FGDs, confidentiality and data transcription/translation/storing/handling.

BMJ Publishing Group Limited (BMJ) disclaims all liability and responsibility arising from any reliance placed on this supplemental material which has been supplied by the author(s)

SPIRIT

STANDARD PROTOCOL ITEMS: RECOMMENDATIONS FOR INTERVENTIONAL TRIALS

SPIRIT 2013 Checklist: Recommended items to address in a clinical trial protocol and related documents*

Section/item	Item No	Description	Yes/No	Page No
**Administrative information**				
Title	1	Descriptive title identifying the study design, population, interventions, and, if applicable, trial acronym	Yes	1
Trial registration	2a	Trial identifier and registry name. If not yet registered, name of intended registry	Yes	2
	2b	All items from the World Health Organization Trial Registration Data Set	Yes	2
Protocol version	3	Date and version identifier	Yes	1-12
Funding	4	Sources and types of financial, material, and other support	Yes	9
Roles and responsibilities	5a	Names, affiliations, and roles of protocol contributors	Yes	1
	5b	Name and contact information for the trial sponsor	Yes	9
	5c	Role of study sponsor and funders, if any, in study design; collection, management, analysis, and interpretation of data; writing of the report; and the decision to submit the report for publication, including whether they will have ultimate authority over any of these activities	N/A	N/A
	5d	Composition, roles, and responsibilities of the coordinating centre, steering committee, endpoint adjudication committee, data management team, and other individuals or groups overseeing the trial, if applicable (see Item 21a for data monitoring committee)	N/A	N/A

**Introduction**				
Background and rationale	6a	Description of research question and justification for undertaking the trial, including summary of relevant studies (published and unpublished) examining benefits and harms for each intervention	Yes	3-4
	6b	Explanation for choice of comparators	Yes	4
Objectives	7	Specific objectives or hypotheses	Yes	4
Trial design	8	Description of trial design including type of trial (eg, parallel group, crossover, factorial, single group), allocation ratio, and framework (eg, superiority, equivalence, noninferiority, exploratory)	Yes	4
**Methods: Participants, interventions, and outcomes**				
Study setting	9	Description of study settings (eg, community clinic, academic hospital) and list of countries where data will be collected. Reference to where list of study sites can be obtained	Yes	4
Eligibility criteria	10	Inclusion and exclusion criteria for participants. If applicable, eligibility criteria for study centres and individuals who will perform the interventions (eg, surgeons, psychotherapists)	Yes	4-5
Interventions	11a	Interventions for each group with sufficient detail to allow replication, including how and when they will be administered	Yes	5
	11b	Criteria for discontinuing or modifying allocated interventions for a given trial participant (eg, drug dose change in response to harms, participant request, or improving/worsening disease)	N/A	N/A
	11c	Strategies to improve adherence to intervention protocols, and any procedures for monitoring adherence (eg, drug tablet return, laboratory tests)	N/A	N/A

	11d	Relevant concomitant care and interventions that are permitted or prohibited during the trial	N/A	N/A
Outcomes	12	Primary, secondary, and other outcomes, including the specific measurement variable (eg, systolic blood pressure), analysis metric (eg, change from baseline, final value, time to event), method of aggregation (eg, median, proportion), and time point for each outcome. Explanation of the clinical relevance of chosen efficacy and harm outcomes is strongly recommended	Yes	5-6
Participant timeline	13	Time schedule of enrolment, interventions (including any run-ins and washouts), assessments, and visits for participants. A schematic diagram is highly recommended (see Figure)	Yes	Figure 1 (Image)
Sample size	14	Estimated number of participants needed to achieve study objectives and how it was determined, including clinical and statistical assumptions supporting any sample size calculations	Yes	6
Recruitment	15	Strategies for achieving adequate participant enrolment to reach target sample size	Yes	6
**Methods: Assignment of interventions (for controlled trials)**				
Allocation:				
Sequence generation	16a	Method of generating the allocation sequence (eg, computer-generated random numbers), and list of any factors for stratification. To reduce predictability of a random sequence, details of any planned restriction (eg, blocking) should be provided in a separate document that is unavailable to those who enrol participants or assign interventions	Yes	6
Allocation concealment mechanism	16b	Mechanism of implementing the allocation sequence (eg, central telephone; sequentially numbered, opaque, sealed envelopes), describing any steps to conceal the sequence until interventions are assigned	Yes	6

Implementation	16c	Who will generate the allocation sequence, who will enrol participants, and who will assign participants to interventions	Yes	6
Blinding (masking)	17a	Who will be blinded after assignment to interventions (eg, trial participants, care providers, outcome assessors, data analysts), and how	Yes	6
	17b	If blinded, circumstances under which unblinding is permissible, and procedure for revealing a participant's allocated intervention during the trial	N/A	N/A
**Methods: Data collection, management, and analysis**				
Data collection methods	18a	Plans for assessment and collection of outcome, baseline, and other trial data, including any related processes to promote data quality (eg, duplicate measurements, training of assessors) and a description of study instruments (eg, questionnaires, laboratory tests) along with their reliability and validity, if known. Reference to where data collection forms can be found, if not in the protocol	Yes	6
	18b	Plans to promote participant retention and complete follow-up, including list of any outcome data to be collected for participants who discontinue or deviate from intervention protocols	Yes	6
Data management	19	Plans for data entry, coding, security, and storage, including any related processes to promote data quality (eg, double data entry; range checks for data values). Reference to where details of data management procedures can be found, if not in the protocol	Yes	7
Statistical methods	20a	Statistical methods for analysing primary and secondary outcomes. Reference to where other details of the statistical analysis plan can be found, if not in the protocol	Yes	7
	20b	Methods for any additional analyses (eg, subgroup and adjusted analyses)	Yes	7
	20c	Definition of analysis population relating to protocol non-adherence (eg, as randomised analysis), and any statistical methods to handle missing data (eg, multiple imputation)	Yes	7

**Methods: Monitoring**				
Data monitoring	21a	Composition of data monitoring committee (DMC); summary of its role and reporting structure; statement of whether it is independent from the sponsor and competing interests; and reference to where further details about its charter can be found, if not in the protocol. Alternatively, an explanation of why a DMC is not needed	N/A	N/A
	21b	Description of any interim analyses and stopping guidelines, including who will have access to these interim results and make the final decision to terminate the trial	N/A	N/A
Harms	22	Plans for collecting, assessing, reporting, and managing solicited and spontaneously reported adverse events and other unintended effects of trial interventions or trial conduct	Yes	9
Auditing	23	Frequency and procedures for auditing trial conduct, if any, and whether the process will be independent from investigators and the sponsor	N/A	N/A
**Ethics and dissemination**				
Research ethics approval	24	Plans for seeking research ethics committee/institutional review board (REC/IRB) approval	Yes	9
Protocol amendments	25	Plans for communicating important protocol modifications (eg, changes to eligibility criteria, outcomes, analyses) to relevant parties (eg, investigators, REC/IRBs, trial participants, trial registries, journals, regulators)	N/A	N/A
Consent or assent	26a	Who will obtain informed consent or assent from potential trial participants or authorised surrogates, and how (see Item 32)	Yes	9
	26b	Additional consent provisions for collection and use of participant data and biological specimens in ancillary studies, if applicable	N/A	N/A
Confidentiality	27	How personal information about potential and enrolled participants will be collected, shared, and maintained in order to protect confidentiality before, during, and after the trial	Yes	9

Declaration of interests	28	Financial and other competing interests for principal investigators for the overall trial and each study site	Yes	9
Access to data	29	Statement of who will have access to the final trial dataset, and disclosure of contractual agreements that limit such access for investigators	Yes	9
Ancillary and post-trial care	30	Provisions, if any, for ancillary and post-trial care, and for compensation to those who suffer harm from trial participation	N/A	N/A
Dissemination policy	31a	Plans for investigators and sponsor to communicate trial results to participants, healthcare professionals, the public, and other relevant groups (eg, via publication, reporting in results databases, or other data sharing arrangements), including any publication restrictions	Yes	4
	31b	Authorship eligibility guidelines and any intended use of professional writers	Yes	8
	31c	Plans, if any, for granting public access to the full protocol, participant-level dataset, and statistical code	N/A	N/A
**Appendices**				
Informed consent materials	32	Model consent form and other related documentation given to participants and authorised surrogates	Yes	Appendix B
Biological specimens	33	Plans for collection, laboratory evaluation, and storage of biological specimens for genetic or molecular analysis in the current trial and for future use in ancillary studies, if applicable	N/A	N/A

*It is strongly recommended that this checklist be read in conjunction with the SPIRIT 2013 Explanation & Elaboration for important clarification on the items. Amendments to the protocol should be tracked and dated. The SPIRIT checklist is copyrighted by the SPIRIT Group under the Creative Commons "Attribution-NonCommercial-NoDerivs 3.0 Unported" license.
