# Supplementary material for: Exploratory randomised trial of face-to-face and mobile phone counselling against usual care for tobacco cessation in Indian primary care: a randomised controlled trial protocol for project CERTAIN
Source: BMJ Open. 2022 Jan 6;12(1):e048628. doi: 10.1136/bmjopen-2021-048628 (PMC8739426; doi:10.1136/bmjopen-2021-048628)
Supplement: Supplementary data [file bmjopen-2021-048628supp002.pdf]

Appendix B: PARTICIPANT INFORMATION SHEET (PATIENT RECRUITMENT: TRIAL)

Project title: Exploratory randomized trial of face to face and mobile phone counselling against usual care for tobacco cessation in Indian primary care

Principal Investigator and ethics secretary contact number and organization:

Dr Rajmohan Panda, MD, MPH<br>Public Health Foundation of India (PHFI)<br>Additional Professor<br>0124-4781400<br>raj.panda@phfi.org	Dr Aastha Agarwal<br>Member Secretary<br>Research Scientist and Assistant Professor<br>Public Health Foundation of India<br>Email: trc- iec@phfi.org<br>Phone: 0124 478 1400

I am __________________ conducting the research on behalf of Dr. Rajmohan Panda, Additional Professor, PHFI. I am conducting a research to test the effectiveness of face to face and mobile phone counselling against usual care for tobacco cessation in Indian primary care settings. This information sheet describes the research and invites you to be a part of it. You can take time to think about your participation in the research. Before you decide, you can talk to anyone about the research. If this information sheet contains words that you do not understand, please feel free to ask me. If you have any other questions too, you can ask me.

Project brief

Tobacco is an important public health issue contributing towards many significant health problem and needs to be prevented. Tobacco cessation counselling has been identified as an important strategy to help people give up using tobacco. The overburdened health professionals and busy hospital out-patient-department provides limited time to the doctors/counsellors to counsel tobacco users. Through this research we aim to develop and test an innovative mobile telephone counselling system to assist tobacco users give up using tobacco.

What is the treatment offered in this trial?

This research may use mobile phone based counselling system using text messages.

Why have you been asked to take part in this research?

You are being invited to take part in this research because you use tobacco. We request you to consider participating as we feel that you will be able to provide us with information that will help in understanding and improving the mobile counselling services which we will then be tested in a trial. If you agree, I would like to ask you about these matters and request you to answer the questions to the best of your knowledge.

Is my participation in this research entirely voluntary?

Your participation in this research is entirely voluntary. It is your choice whether to participate or not. You do not have to take part in this research if you do not wish to do so, and choosing to participate will not affect your treatment or treatment-related evaluations in any way.

If you do take part, the interview will take about 25 minutes to 40 minutes and I want to assure you that the information that you provide will be kept confidential. Your name or other information that could identify you will not appear in research record or report. You are free to withdraw from this research at any time should you change your mind.

Do you have any questions?

What are the risks of my participation in this research?

We are asking you to share with us some information related to your health and habits, and you may feel uncomfortable talking about some of the topics. You do not have to answer any question you do not want to answer. Nor do you need take part in the intervention or interview if you don't wish to do so, and that is all fine. You do not have to give us any reason for not responding to any question or for refusing to take part in the interview. The information that you give is confidential and will not be shared by any one in any manner that can identify you.

What are the benefits of my participation in this research?

By participating in the research you are contributing in the development of a counselling mechanism which will help people (including yourself) quit tobacco use in the future. This will have direct benefit to your health and that of other people using tobacco.

Will the information I provide be kept private and confidential?

We will ensure your privacy is maintained during the interviews. This will be ensured by conducting one interview at a time in a room in the health facility. We will not share the information you provide with anyone other than the research team. The information so collected from this research will be kept private in a secure manner and none of your personal details will appears on this information. Rather it will be allocated a number which only the researchers will be able to associate the number with you. The information provided by you hence will not be attributed to you by name. The knowledge that we get from this research will be shared with you and your community before it is made widely available to the public.

Do I have the right to refuse or withdraw from the research?

You do not have to take part in this research if you do not wish to do so, and choosing to participate will not affect your treatment or treatment-related evaluations in any way. You may stop participating in the interview at any time that you wish without your treatment being affected.

Respondent agrees for Interview…………………1 → BEGIN THE INTERVIEW AFTER

Respondent does not agree for interview…………2 → END

CONSENT FORM (PATIENT RECRUITMENT: TRIAL)

Project title: Exploratory randomized trial of face to face and mobile phone counselling against usual care for tobacco cessation in Indian primary care.

Name of Researcher/ Secretary Ethics committee:

Dr Rajmohan Panda, MD, MPH<br>Public Health Foundation of India (PHFI)<br>Additional Professor<br>0124-4781400<br>raj.panda@phfi.org	Dr Aastha Agarwal<br>Member Secretary<br>Research Scientist and Assistant Professor<br>Public Health Foundation of India<br>Email: trc- iec@phfi.org<br>Phone: 0124 478 1400

Please tick the box

1. I confirm that I have read the information sheet for the above research. I have had the opportunity to consider the information, ask questions and have had these answered satisfactorily. ☐
2. I understand that my participation is voluntary and that I am free to withdraw at any time without giving any reason, without my medical care or legal rights being affected. ☐
3. I understand that the information collected about me will be used to support other research in the future, and may be shared anonymously with other researchers. ☐
4. I agree to my primary care practitioner (PCP) being informed of my participation in the research. / I agree to my primary care practitioner being involved in the research, including any necessary exchange of information about me between my PCP and the research team. ☐
5. I agree to take part in the above research. ☐

______________________________ ____________

Name and Signature (Participant) Date

______________________________ ____________

Name and Signature (Consent Taker) Date
