# Supplementary material for: Exploratory randomised trial of face-to-face and mobile phone counselling against usual care for tobacco cessation in Indian primary care: a randomised controlled trial protocol for project CERTAIN
Source: BMJ Open. 2022 Jan 6;12(1):e048628. doi: 10.1136/bmjopen-2021-048628 (PMC8739426; doi:10.1136/bmjopen-2021-048628)
Supplement: Author's manuscript [file bmjopen-2021-048628.draft_revisions.pdf]

BMJ Open is committed to open peer review. As part of this commitment we make the peer review history of every article we publish publicly available.

When an article is published we post the peer reviewers' comments and the authors' responses online. We also post the versions of the paper that were used during peer review. These are the versions that the peer review comments apply to.

The versions of the paper that follow are the versions that were submitted during the peer review process. They are not the versions of record or the final published versions. They should not be cited or distributed as the published version of this manuscript.

BMJ Open is an open access journal and the full, final, typeset and author-corrected version of record of the manuscript is available on our site with no access controls, subscription charges or pay-per-view fees (http://bmjopen.bmj.com).

If you have any questions on BMJ Open's open peer review process please email info.bmjopen@bmj.com

BMJ Open

Exploratory randomized trial of face to face and mobile phone counselling against usual care for tobacco cessation in Indian primary care: a randomized controlled trial protocol for project CERTAIN.

Journal:	*BMJ Open*
Manuscript ID	bmjopen-2021-048628
Article Type:	Protocol
Date Submitted by the Author:	05-Jan-2021
Complete List of Authors:	Panda, Rajmohan; Public Health Foundation of India, research<br>Omar, Rumana; University College London,<br>Hunter, Rachael; University College London, Research Dept of Primary Care and Population Health<br>Prabhu, Rajath R; Public Health Foundation of India, research<br>Mishra, Arti; Independent Consultant, Research<br>Nazareth, Irwin; UCL, Primary Care and Population Health
**Primary Subject Heading**:	
Secondary Subject Heading:	
Keywords:	Protocols & guidelines < HEALTH SERVICES ADMINISTRATION & MANAGEMENT, PRIMARY CARE, QUALITATIVE RESEARCH, PUBLIC HEALTH

SCHOLARONE™
Manuscripts

Running Title: Counselling intErvention foR smokeless Tobacco cessAtion in INdian primary care: The CERTAIN study

Title: Exploratory randomized trial of face to face and mobile phone counselling against usual care for tobacco cessation in Indian primary care: a randomized controlled trial protocol for project CERTAIN.

Correspondence to: Dr Rajmohan Panda, raj.panda@phfi.org, Public Health Foundation of India, Plot Number 47, Sector 44, Gurugram, Haryana, India

Rajmohan Panda, Research Division, Public Health Foundation of India, Plot Number 47, Sector 44, Gurugram, Haryana, India

Rumana Omar, Department of Statistical Science, University College London, Gower Street, London, WC1E 6BT, United Kingdom

Rachael Hunter, Department of Primary Care and Population Sciences, University College London, Gower Street, London, WC1E 6BT, United Kingdom

Rajath R Prabhu, Research Division, Public Health Foundation of India, Plot Number 47, Sector 44, Gurugram, Haryana, India

Arti Mishra, Research, Independent Consultant

Irwin Nazareth, Department of Primary Care and Population Sciences, University College London, Gower Street, London, WC1E 6BT, United Kingdom

Word Count: 3883 (excluding title page, abstract, references, figures and tables)

Keywords/Phrases: tobacco cessation; smokeless tobacco; mobile counselling; primary care; formative research; exploratory randomized controlled trial

For peer review only

For peer review only - http://bmjopen.bmj.com/site/about/guidelines.xhtml

Abstract

Introduction: There is an overwhelming use of smokeless tobacco products in the Indian sub-continent and little awareness of its health hazards when compared to smoked tobacco. We hypothesize that mobile phone counselling will be a feasible, affordable and effective for smokeless tobacco cessation intervention in India. This paper describes the protocol of the development and conduct of an exploratory trial before progression to a full randomized trial.

Methods and Analysis: An exploratory randomized controlled trial will be conducted in primary care clinics in the state of Odisha, India. A total of 250 tobacco users will be recruited to the study (125 in each arm). Participants in the intervention arm will receive routine care together with a face to face counselling intervention followed by advice and reminder mobile messages. The control arm will receive routine care. All recruited participants will be followed-up for three or six months depending on the COVID-19 situation, from the first counselling session. Formative research relating to the development and finalisation of the intervention will be conducted before the exploratory trial.

Ethics and Dissemination: Ethical approvals were obtained from the Institutional Ethics Committee of Public Health Foundation of India, Health Ministry's Screening Committee – ICMR, Odisha State Ethics Board and also from University College London Research Ethics Committee, UK.

Trial Registration Number: CTRI Registration Number CTRI/2019/05/019484; dated May 31, 2019

Article Summary:

Strengths and limitations of this study:

- Evidence on the effectiveness of smokeless tobacco cessation intervention in primary care scares. The results of this study will inform on the feasibility of conducting such trials in the future.
- As mobile phones have become a regular part of the daily lives across the globe, incorporating this technology in routine clinical practice, will be time-efficient as the messages can be delivered anywhere, at fixed times, and directly to the participant with negligible direct contact whilst also ensuring privacy.
- The impact of this research will also be that the training of health professional will lead to an improved understanding of the delivery of low-cost tobacco cessation interventions in primary care.
- The study findings cannot be generalised to other healthcare settings as this study is limited to urban public health setting (Urban Public Health Centres).

For peer review only - http://bmjopen.bmj.com/site/about/guidelines.xhtml

Introduction

Tobacco use is responsible for almost eight million deaths each year or a death every six seconds.[1,2] About 80% of 1.3 billion tobacco consumers across the globe live in Low-and Middle-Income Countries (LMICs).[1] In LMICs like India, the tobacco problem is very complex as the country has a diverse population with a mixture of different cultures, religions, and practices. The majority of people use a variety of tobacco products – combustible, non-combustible, or both. As per the Global Adult Tobacco Survey 2 (GATS 2), 28.6% of the adult population in India consumes tobacco (10.7% of smoking and 21.4% of smokeless) making it the second-largest consumer in the world.[3]

Widespread use of smokeless tobacco (SLT) products is seen in countries like India and Bangladesh such that 232 of 248 million SLT users worldwide (i.e. 21 countries) are from these two countries.[4] SLT products such as "*tambaaku*", "*gurkha*", "*zarda*", "*khaini*", "*mawa*" and "*pan masala*" are widely used in many states in India as SLT consumption is culturally accepted and is considered as a common practice. [5] SLT use is double than that of combustible tobacco in India, making it single-most common form of tobacco used by both men and women (29.6% and 12.8% respectively). [5] According to GATS 2, Odisha was found to be one of the states where the percentage of adults who consume SLT products (43%) was much higher than the national average (21.4%).[6] There is a misconception that SLT is less harmful, despite strong evidence on the carcinogenicity of SLT[7] in the oral cavity, pharynx, and oesophagus that account for a large proportion of tobacco-related cancers in India.[8] Yet few users are aware of the health risks of SLT compared to smoked tobacco.

Primary care health professionals, first contact for patients accessing help, play a key role in health education on hazards of tobacco and are best placed to counsel against tobacco use and promote cessation. Studies have shown that brief interventions like tobacco cessation counselling by healthcare providers are effective in quitting tobacco use.[9,10] A systematic review conducted on SLT cessation intervention included studies conducted in both high-income countries (HICs) and LMICs showed that behavioural interventions are effective in achieving cessation.[11] However, overburdened primary care physicians in India have limited time for counselling.[2,12,13] It is also difficult to get tobacco users to attend follow-up visits as they would incur a loss of income to travel to primary care clinics.[12]

In India, the mobile phone has wide coverages in both rural and urban settings. India with 1173.7 million mobile phone users is second-largest user base in the world.[14] The mobile phone subscribers in India increased from 1187 million in June 2019 to 1195.2 million by September 2019.[14] A meta-analysis on the effectiveness of telephone counselling for smoking tobacco cessation that included 104 trials showed significant but modest effect sizes in HICs.[15] Effectiveness increases with treatment intensity.[16] A review of 26 trials on smoking cessation demonstrated that automated text messaging interventions were more effective than minimal smoking cessation support (RR 1.54, 95% CI 1.19 to 2.00; I2 = 71%; 13 studies) and that the text messaging added to other smoking cessation interventions was more effective than other smoking cessation interventions individually (RR 1.59, 95% CI 1.09 to 2.33; I2 = 0%, 4 studies).[17] Another meta-analysis of 13 trials reported smoking quit rates with text messaging intervention were 35% higher than quit rates for controls (OR = 1.35, 95% CI = 1.23, 1.49).[18] Similar review studies from HICs suggest that such intervention can increase the chance of quitting smoking tobacco from 39% to 80%.[19,20] Studies on SLT cessation that included randomized controlled trials conducted in HICs as well as some LMICs showed that behavioural cessation interventions led to quit rates between 9% to 54% at six months.[11,21] However, there are little data on efficacy and effectiveness of such intervention combined with mobile phone-based counselling in LMIC like India. Improved participation in tobacco cessation programmes through the use of mobile phone technology can have a substantial impact on a population at risk even with a small effect size.[22]

For peer review only - http://bmjopen.bmj.com/site/about/guidelines.xhtml

There is an urgent need to test the effectiveness of the use of mobile phone messages for SLT cessation in the Indian primary care setting.[23] We hypothesize that a face-to-face intervention offered by a tobacco counsellor together with low-cost mobile-based counselling has the potential to deliver a high-quality cessation intervention to tobacco users.

Aims and Objectives

Our overarching aim is to evaluate within a randomized controlled trial, the clinical effectiveness (as measured by tobacco cessation) and cost-effectiveness of a complex intervention of a face to face counselling coupled with mobile phone messaging delivered to SLT users visiting Indian primary care clinics in addition to routine care provided by the primary care professional against routine care alone. Prior to undertaking a full trial, we aim to develop and finalise the intervention and then test its acceptability and feasibility prior to running a full randomized controlled trial. Specific objectives of the study are to:

1. Finalise the development of intervention and usual care and to detail their delivery in the study manuals.
2. Assess within an exploratory randomized trial, the proportion of all primary care tobacco user attenders approached to take part in the trial that offer consent to be randomized.
3. Assess within the exploratory randomized trial, the proportion of those who are randomized to intervention that complies with the intervention.
4. Assess within the exploratory randomized trial, the proportion of those who are randomized on whom the follow up data is collected.
5. Assess within the exploratory randomized controlled trial, the proportion of missing data on all research measures made at baseline and three or six months that will be eventually used to assess the primary and secondary outcomes.

Methodology

Patient and Public Involvement

Patient and public were not involved in formulation of study hypothesis, research question, outcome measures or experimental design. The results and additional information of this study will be provided to study participants on request.

Study Design

Study will be conducted in two different phases. The first phase is a formative phase and the second phase is an exploratory randomized controlled trial. The duration of study will be 18 months.

Study Setting

The study will be conducted in primary care clinics / urban primary health centres (UPHCs). All UPHCs in Berhampur city of Ganjam district of Odisha will be selected for this study.

Ethical Approval and Consent

Ethics approval has been taken from the Institutional Ethical Committee at Public Health Foundation of India (PHFI) (ref: TRC-IEC-391/19; dated May 29, 2019). At the national level, ethical clearance has been obtained from the Health Ministry's Screening Committee (HMSC), led by Indian Council of Medical Research (ICMR) (ref: 2019-3581; dated December 11, 2019). Also, local level approval was obtained from the Odisha State Ethics Board (ref: 191/PMU/187/17; dated November 14, 2019). In the UK, ethical clearance was obtained by the UCL Research Ethics Committee (ref: 5686/001, dated October 1, 2019). The study has been registered at Clinical Trials Registry India (reference number CTRI/2019/05/019484). All the participants (participating in both the phases in this study) will be

For peer review only - http://bmjopen.bmj.com/site/about/guidelines.xhtml

provided with a participant information sheet (PIS), providing details of the study. Following which, voluntary written consent for participation will also be taken from them. All the data that will be collected in the study will be stripped of any personal identifiers and the data will be stored in PHFI's data repository. The data will only be accessible to the principal investigator and trial team analysing the data.

Study phase I: Formative Research

Qualitative research methods will be used in this phase. In-Depth Interviews (IDIs) will be conducted with SLT users as well as primary care physicians present in UPHCs and Focus Group Discussions (FGDs) will be conducted with tobacco cessation counsellors. The formative research study will be based on a maximum variation sampling.[24] A maximum variation sample will be constructed by identifying key dimensions of variations and then finding participants that vary from each other as much as possible. This sampling will yield detailed descriptions of each respondent, useful for documenting uniqueness, and this will allow researchers to identify common themes that are evident across the heterogeneous group of study participants. The variation will cover the socio-demographic characteristics of the study participants. The formative phase will assist in finalising two components of the intervention, a single ten minutes face to face counselling that will be delivered by a tobacco cessation counsellor and a mobile phone intervention (regular messages) which will be offered three times a week over three or six-month duration depending on the COVID-19 situation. The formative research will be conducted in three sequential phases.

Phases in Formative Research:

In Phase I, IDIs will be conducted with a purposive sample of male (n=15) and female (n=15) SLT users visiting UPHCs during the study period. IDIs will also be conducted with primary care physicians (n=5) present at these UPHCs. Participants' views on the detail of text messages will be explored. These views will inform the final text messages deemed suitable for tobacco cessation support. The messages will be tailored to users' health conditions and will use local terms, dialects and opinions of various stakeholders.

In Phase IIA, two FGDs will be conducted each with six primary care staff (ANM, ASHA, pharmacists, staff nurse). Focus groups will explore the perspective of participants' regarding the preliminary content and structure of messages, use of tailored messages addressing the risk of tobacco for specific diseases, associated comorbidities, risks of tobacco usage in these comorbid conditions, readiness to quit stage and withdrawal symptoms.

In Phase IIB, developed text messages will undergo validation. The validation study will be conducted with SLT users visiting UPHCs. Each will be given a validation tool (developed specifically for this study) which will be used to score each of the newly developed messages. The messages that get the highest score will be adopted as a part of the intervention. IDIs will also be conducted with these tobacco users to gain their views and opinions regarding developed text messages.

Phase III, will be conducted at two-time points (i.e., mid-line and end-line) in the trial follow-up period. At mid-line (1.5/3 months), IDIs will be conducted with SLT users to assess the appropriate engagement of the text messages. At end of three or six-months, IDIs will be conducted with SLT users who complete the follow-up period as well as with the SLT users who drop out of the study. Perceptions about appropriate engagement and satisfaction with text messages will be captured at this point.

For peer review only - http://bmjopen.bmj.com/site/about/guidelines.xhtml

The IDIs and FGDs will be conducted by trained qualitative research team members. Each team will have a moderator and note-taker. All interviews will be audio-recorded (with participants' consent) and transcribed. The transcriptions will be analysed using a qualitative framework analysis method. All transcripts will be reviewed and coded by two members of the research team to minimize subjective interpretations. We will use Atlas.ti (Version 8) software[25] to evaluate the data.

Training of qualitative research team: All members of the qualitative research team participating in the study at each UPHCs will be trained. The training of qualitative researchers will include how to introduce the study to potential participants, obtaining verbal and written consent, conducting IDIs and FGDs, confidentiality and data transcription/translation/storing/handling.

Training of tobacco cessation counsellors delivering the intervention: Counsellors at UPHCs are allied healthcare workers and will be trained by the members of the research team to deliver counselling intervention. They will not be involved in data collection or data analysis. Their training will be done using a standardized training manual (hardcopy of study manual will be provided to these counsellors) to maintain the fidelity of the intervention.

Study phase II: Exploratory randomized controlled trial

The second phase in this study will be an exploratory, parallel-group, randomized controlled trial, with pre-and post-test assessments.

Study population: The study participants are adult (above 18 years) SLT users who visit UPHCs in Berhampur city, Odisha. To be included, participants must be current SLT users, ages 18 years and above, have a mobile phone with a valid contact number and consent to participate in the study.

Participants who are below 18 years of age, who do not have the mental capacity to consent, who suffer from illness limiting their adherence and follow-up in the study, and who do not provide consent to participate will be excluded.

Randomization: Randomization will be done at the level of individual eligible participants who have provided informed consent. Randomization will be stratified by study practice site (UPHCs) using random permuted blocks of varying block sizes from 4 to 10, and a 1:1 allocation to intervention or control arm. A randomization list will be prepared by an independent statistician (from PRIMENT CTU, UCL) not involved with this study. This list will be mailed to an independent staff member at the study site who will be responsible for the allocation of participants to the respective intervention arm. Each case will be assigned a participant identification number. Treatment allocation for the recruited participants will be emailed to the treatment centre.

The trial will ensure a clear separation between staff that collect outcome data and those that deliver the intervention. Staff collecting outcome data will be blinded to the group assignment. None of the intervention staff, primary care physicians and behavioural counsellors will collect outcome data. All investigators, staff, and participants will be kept masked to outcome measurements and any preliminary results emerging from the trial.

The arm of the study and Intervention: The intervention arm includes routine care (component 1) along with a single ten minutes face to face intervention based on 5As approach (component 2) and followed by mobile phone counselling (component 3). The control arm will be provision of routine care alone (i.e. Component 1).

For peer review only - http://bmjopen.bmj.com/site/about/guidelines.xhtml

CERTAIN Intervention: The intervention will be a combination of the following components -

1) Component one or delivery of “routine care” as delivered by a primary care physician over one to two minutes. This component is common to both intervention and control arms of the study. This is based on ‘Ask’ and ‘Advice’. Under ‘Ask’, the physician will ask about consumption of SLT and under ‘Advice’, the physician will inform and advice participants about the benefits of complete abstinence in a clear personalized manner.

2) Component two will include a single 10 minutes face to face intervention delivered by a practice-based counsellor. This component will include brief standardized advice provided to participants based on 5A’s approach[16] of tobacco cessation. Under this approach the counsellor will ‘Ask’ the participants if they use SLT; ‘Advise’ them on importance of quitting (following the same script of component 1); ‘Assess’ their willingness to ‘quit now’; offer them ‘Assistance’ in the form of Nicotine Replacement Therapy (NRT) and/or provide them with a referral for behavioural support; and ‘Arrange’ a follow-up to check on progress. This will conclude with their enrolment to receive Component three – mobile phone intervention.

3) Component three will also follow 5A’s approach to tobacco cessation. The intervention will include an initial message after 48 hours of recruitment, to remind them to quit tobacco followed by messages occurring three times a week for the next three or six-months depending on the COVID-19 situation. Each follow-up message will offer behavioural messages to support quitting; information about health risks of tobacco use and benefits of quitting, and advice on withdrawal symptoms and coping strategies.

Control Arm: The control group will consist of only component one or delivery of “routine care” delivered by a primary care physician as described above.

Outcome Measurement: Outcomes will be assessed at baseline on recruitment to the study and the end of three or six-months depending on the COVID-19 situation. At baseline, socio-demographic details such as age, sex, educational level, marital status, religion, community, the economic status will be recorded. Variables related to tobacco consumption will be recorded including, number of SLT products per day (frequency), age of initiation, number of previous cessation attempts, and longest period of cessation. The data on quit attempts, the status of tobacco use, and challenges will be recorded during baseline and end-line. The biochemical validation of self-reported tobacco abstinence for seven days will be assessed using salivary cotinine at the end of three or six months. The rapid test kit works through visual interpretation of colour developed on the kit and is instant in processing.[26,27] Participants will be approached by the trial team members to provide with a small sample of saliva which will be collected through passive drooling. Collection, transportation and disposition of the biological samples and the kit will be managed at the study site by the site coordinator and trial team. Cotinine is a preferred marker for tobacco use because cotinine stays in the body much longer than nicotine. Presence of cotinine in saliva or urine is a reliable indicator for tobacco use. Cotinine in vivo has half-life of about twenty hours and lasts for about 3-4 days in the saliva and urine.[28,29]

In case of any threat to any on-going medical procedures, we will discontinue the intervention and refer the participant to a tobacco cessation clinic. In case of any mental health pathology identified during the tobacco cessation counselling, the treatment will be discontinued and participant will be referred to primary care practitioner who will then refer them on to a psychiatrist for further follow-up.

For peer review only - http://bmjopen.bmj.com/site/about/guidelines.xhtml

Data Management: Baseline and end-line questionnaire response data will be collected at the study site and will be entered electronically in the database developed in MS Access (Microsoft Office 2019). Data quality of baseline and end-line data will be monitored by site personnel for completeness, validity and integrity. In case the study personnel find any incomplete or inconsistent response, S/he will verify with the study participant over the call and will update the response in the database if needed.

Outcomes: The main outcomes expected from this study include the:

1. Development of the interventions and usual care and detailing them in a manual;
2. Proportion of all the tobacco users approached who consent to randomization; and
3. Proportion of those randomized on whom follow-up data are collected at three or six months;

As a part of the feasibility work we will also assess the feasibility of assessing outcomes in the main trial as listed below:

Primary Outcomes

- Three or six-month "self-reported tobacco abstinence" of seven days confirmed by a salivary cotinine test.

Secondary Outcomes

- Proportion of those randomized who comply with the intervention.
- Self-reported motivation and intention to quit.
- Cost-effectiveness as cost per disability-adjusted life year (DALY) averted.

Sample Size

A total of 250 individuals (125 individuals per group) is required to estimate an anticipated participant recruitment rate of 50% with a 95% confidence interval (CI) of 44% to 57% and a six-month attrition rate of 20% with 95% CI of 15% to 25%.[30]

Data Analysis

Statistical Analysis: Data will be analysed using STATA version 16 (StataCorp. 2019. *Stata Statistical Software: Release 16*. College Station, TX: StataCorp LLC). Participant characteristics will be summarized using mean and standard deviation [SD] or median and interquartile range for continuous variables, and number & percentages for the categorical variables. The recruitment rate and proportions lost to follow-up will be estimated with 95% confidence intervals. Applicable regression models will be used to estimate the intervention effect with 95% confidence intervals for the specified outcome (tobacco abstinence and self-reported motivation and intention to quit) as part of the secondary analyses. Extent of missing data for each variable and the percentage of participants adhering to the intervention will be reported.

Economic Evaluation: The aim will be to evaluate the feasibility of calculating the cost-effectiveness of routine care along with intervention compared to routine care alone from an Indian health care cost perspective over three or six months. This will include the level of data completeness of self-completed questionnaires of tobacco-related health care resource use, estimation of the intervention and routine care costs. The feasibility of calculating cost per DALY averted in the main trial will also be evaluated in addition to data requirements for a decision model to evaluate lifetime cost-effectiveness of the intervention.

For peer review only - http://bmjopen.bmj.com/site/about/guidelines.xhtml

Discussion

To the best of our knowledge, this is the first study evaluating a complex low-cost intervention up of two components i.e., face-to-face counselling along with mobile phone messaging counselling delivered to SLT users in India and low resource settings for over three or six months. The first component of the intervention is based on 5A's approach which has shown effectiveness in previous studies conducted in primary care for tobacco cessation.[31] However, such a brief intervention may have a limited effect if the person is not ready to quit and due to various other factors associated with difficulty in quitting tobacco.[32] However, follow up quitting advise to tobacco users can increase quit attempts and quit rates[31] and we will be achieving this through the second component of our intervention i.e., the mobile phone messaging. These are widely used in the developed world and research on smoking cessation suggests that they can increase tobacco quitting by 25-50%.[20] But till date there has been no evaluations of its effectiveness in low resource settings and in primary care. The development and delivery of comprehensive, tailored primary care behavioural and mobile phone health technology interventions will enhance the understanding of low-cost long-term prevention intervention for tobacco cessation, will address issues around the evaluation and implementation in low resource settings and will also be addressing risk factors for non-communicable diseases (NCDs).

Recent evidence has emerged on the probable association of tobacco use with novel coronavirus disease.[33,34] The use of SLT often involves hand to mouth contact that can promote the spread of COVID-19. Another behaviour that can spread infections associated with the use of SLT (i.e. chewing tobacco) is spitting of or projection of excess saliva produced during the chewing process.[35] Whilst, we continue to struggle with coronavirus infection control we have an opportunity to expand cessation intervention specifically designed for SLT use and also promote good infection control practice.

Research Impact: In this study, SLT cessation intervention will be developed using an innovative method. The method comprises of the formative research phase, where the SLT cessation intervention will be developed; and the exploratory randomized controlled trial phase, where evaluation of the developed SLT cessation intervention will be done. The evidence generated from this exploratory study will ascertain acceptability and feasibility of SLT cessation intervention in users of SLT and will inform the conduct of a larger multi-centric trial across several countries. Findings from this study will give insights to researchers for designing similar studies and appropriate interventions in tobacco cessation and NCDs. Since the research in this area is in its infancy in LMIC, this work will provide an impetus for researchers working in the area of tobacco cessation to generate new evidence. They will be in a position to adapt methodologies employed by us and utilise the tools developed and piloted in this study.

Trial status: The current COVID-19 pandemic has delayed the process of recruitment to the randomized controlled trial as out-patient departments of the primary care (from where the participants will be recruited) have been shut down and are operating with a limited staff. We anticipate the OPDs will be fully functional from March 2021 and thus, we plan to start recruitment by April 2021. Study is expected to be completed by June 2021.

Contributions: All authors contributed to the manuscript. RP (PI of the study – India) and IN (PI of the study – UK) designed the study, provided guidance to the writing of the paper, reviewed and finalized the paper. RO provided statistical guidance. RH provided inputs on designing economic evaluation section of the study. AM provided inputs on designing the formative research section of the study.

For peer review only - http://bmjopen.bmj.com/site/about/guidelines.xhtml

RRP reviewed and helped in writing the paper. All authors provided the critical feedback and helped in shaping the manuscript.

Funding statement: This research work is funded by the Joint Global Health Trials Initiative MRC/DfID/Wellcome Trust (ref: MR/P021166/1)

Sponsor: This research work is sponsored by University College London, Gower Street, London, WC1E 6BT, United Kingdom

Competing interests: None declared

Ethics and Dissemination: Ethical approvals were obtained from Institutional Ethics Committee – Public Health Foundation of India (ref: TRC-IEC-391/19; dated May 29, 2019), Health Ministry's Screening Committee – ICMR (ref: 2019-3581; dated December 11, 2019), Odisha State Ethics Board (dated November 14, 2019) and also from University College London (UCL) Research Ethics Committee, UK (ref: 5686/001, dated October 1, 2019).

Data sharing statement: Data sharing not applicable as no datasets generated and/or analysed for this study.

References

1. World Health Organization. Tobacco [Internet]. [cited 2020 Jul 20]Who.int. World Health Organization: WHO; 2018. Available from: https://www.who.int/news-room/fact-sheets/detail/tobacco

2. Amit S, Bhambal A, Saxena V et al. Tobacco cessation and counseling: a dentists' perspective in Bhopal city, Madhya Pradesh. Indian J Dent Res Off Publ Indian Soc Dent Res. 2011 Jun;22(3):400–3.

3. GATS 2 Global Adult Tobacco Survey [Internet]. [cited 2020 Jul 20] Available from: https://www.who.int/tobacco/surveillance/survey/gats/GATS_India_2016-17_FactSheet.pdf

4. GATS Atlas Online [Internet]. gatsatlas.org. [cited 2020 Jul 20]. Available from: http://gatsatlas.org/pdf/mobile/index.html#p=38

5. Murthy P, Subodh BN, Sinha D, et al. Smokeless tobacco (SLT) use and cessation in India: Lessons from user and health care provider perspectives. Asian J Psychiatry. 2018 Feb;32:137–42.

6. GATS 2 Global Adult Tobacco Survey [Internet]. [cited 2020 Jul 20]. Available from: https://tiss.edu/uploads/files/Odisha.pdf

7. Monika S, Dineshkumar T, Priyadharini S, et al. Smokeless Tobacco Products (STPs) Harbour Bacterial Populations with Potential for Oral Carcinogenicity. Asian Pac J Cancer Prev. 2020 Mar 1;21(3):815–24.

8. Raute LJ, Sansone G, Pednekar MS, et al. Knowledge of health effects and intentions to quit among smokeless tobacco users in India: findings from the International Tobacco Control Policy Evaluation (ITC) India Pilot Survey. Asian Pac J Cancer Prev APJCP. 2011;12(5):1233–8.

9. Gordon JS, Andrews JA, Crews KM, et al. The 5A's vs 3A's plus proactive quitline referral in private practice dental offices: preliminary results. Tob Control. 2007 Aug;16(4):285–8.

For peer review only

For peer review only - http://bmjopen.bmj.com/site/about/guidelines.xhtml

10. Gordon JS, Andrews JA, Albert DA, et al. Tobacco Cessation via Public Dental Clinics: Results of a Randomized Trial. Am J Public Health. 2010 Jul;100(7):1307–12.

11. Nethan ST, Sinha DN, Chandan K, et al. Smokeless tobacco cessation interventions: A systematic review. Indian J Med Res. 2018 Apr 1;148(4):396.

12. Champassak SL, Goggin K, Finocchario-Kessler S, et al. A qualitative assessment of provider perspectives on smoking cessation counselling. Journal of Evaluation in Clinical Practice. 2014 Jun 1;20(3):281–287.

13. Jradi H. Awareness, practices, and barriers regarding smoking cessation treatment among physicians in Saudi Arabia. J Addict Dis. 2017 Jan 2;36(1):53–9.

14. Bhawan M. The Indian Telecom Services Performance Indicators, July – September, 2019 [Internet]. [cited 2020 Jul 20] 2020 Jan. Available from: https://trai.gov.in/sites/default/files/PIR_08012020_0.pdf

15. Matkin W, Ordóñez-Mena JM, Hartmann-Boyce J. Telephone counselling for smoking cessation. Cochrane Database Syst Rev. 2019 May 02 [Internet].[cited 2020 May 24];(5). Available from: https://www.cochranelibrary.com/cdsr/doi/10.1002/14651858.CD002850.pub4/full

16. Fiore M, United States. Tobacco Use And Dependence Guideline Panel. Treating tobacco use and dependence : 2008 update. Darby, Pa.: Diane Pub. Co; 2009.

17. Whittaker R, McRobbie H, Bullen C, et al. Mobile phone text messaging and app-based interventions for smoking cessation. Cochrane Database Syst Rev. 2019 Oct 22;10:CD006611

18. Spohr SA, Nandy R, Gandhiraj D, et al. Efficacy of SMS Text Message Interventions for Smoking Cessation: A Meta-Analysis. J Subst Abuse Treat. 2015 Sep;56:1–10.

19. Whitehouse E, Lai J, Golub JE, et al. A systematic review of the effectiveness of smoking cessation interventions among patients with tuberculosis. Public Health Action. 2018 Jun 21;8(2):37–49.

20. Stead LF, Hartmann-Boyce J, Perera R, et al. Telephone counselling for smoking cessation. Cochrane Database Syst Rev. 2013 Aug 12;(8):CD002850.

21. Messina J, Freeman C, Rees A, et al. Smokeless tobacco: South Asians -Reviews 1 and 2 - Effectiveness and contextual factors Systematic review of effectiveness of smokeless tobacco interventions for South Asians and a review of contextual factors relating to smokeless tobacco among South Asian users and health care providers [Internet]. [cited 2020 Jul 20]. Available from: https://www.nice.org.uk/guidance/ph39/documents/smokeless-tobacco-cessation-south-asians-evidence-reviews-1-and-22

22. Baskerville NB, Struik LL, Hammond D, et al. Effect of a mobile phone intervention on quitting smoking in a young adult population of smokers: randomized controlled trial study protocol. JMIR Res Protoc. 2015 Jan 19;4(1):e10.

23. IndianJMedRes1406707-3538149_094941.pdf [Internet]. [cited 2020 Jul 20]. Available from: http://www.ijmr.org.in/temp/IndianJMedRes1406707-3538149_094941.pdf

24. Palinkas LA, Horwitz SM, Green CA, et al. Purposeful sampling for qualitative data collection and analysis in mixed method implementation research. Adm Policy Ment Health. 2015 Sep;42(5):533–44.

For peer review only - http://bmjopen.bmj.com/site/about/guidelines.xhtml

25. ATLAS.ti 8 Windows [Internet].[cited 2020 Jul 20] ATLAS.ti. Available from: https://atlasti.com/product/v8-windows/

26. Jarvis MJ, Tunstall-Pedoe H, Feyerabend C, et al. Comparison of tests used to distinguish smokers from nonsmokers. Am J Public Health. 1987 Nov;77(11):1435–8.

27. Benowitz NL. Biomarkers of environmental tobacco smoke exposure. Environ Health Perspect. 1999 May;107(Suppl 2):349–55.

28. Honarmand M, Nakhaee A, Moradi M. Comparison of Salivary Cotinine Concentrations in Male Smokers and Smokeless Tobacco Users. Asian Pac J Cancer Prev APJCP. 2018;19(5):1363–6.

29. What to Know if You Have to Take a Nicotine Test [Internet].[cited 2020 Jul 20] WebMD. Available from: https://www.webmd.com/smoking-cessation/taking-nicotine-test#1

30. Sample Size Tables for Clinical Studies, 3rd Edition | Wiley [Internet]. Wiley.com. [cited 2020 Oct 7]. Available from: https://www.wiley.com/enus/Sample+Size+Tables+for+Clinical+Studies%2C+3rd+Edition-p-9781444357967

31. Mahapatra S, Panda R. Effectiveness of face-to-face counseling with follow-up in primary care settings in India. Tobacco Induced Diseases. 2018 Mar 1;16(1).

32. Bailey SR, Stevens VJ, Fortmann SP, et al. Long-Term Outcomes from Repeated Smoking Cessation Assistance in Routine Primary Care. Am J Health Promot AJHP. 2018 Sep;32(7):1582–90.

33. Mishra GA, Pimple SA, Shastri SS. An overview of the tobacco problem in India. Indian J Med Paediatr Oncol Off J Indian Soc Med Paediatr Oncol. 2012;33(3):139–45

34. Kaur J, Rinkoo AV. Public Health Perspectives of Smokeless Tobacco and Areca Nut Use in the COVID-19 Era. Nicotine & Tobacco Research. 2020 May 8;

35. WHO. Q&A: Tobacco and COVID-19 [Internet]. www.who.int. 2020 [cited 2020 Jul 20]. Available from: https://www.who.int/news-room/q-a-detail/q-a-on-tobacco-and-covid-19

For peer review only

For peer review only - http://bmjopen.bmj.com/site/about/guidelines.xhtml

Study Overview

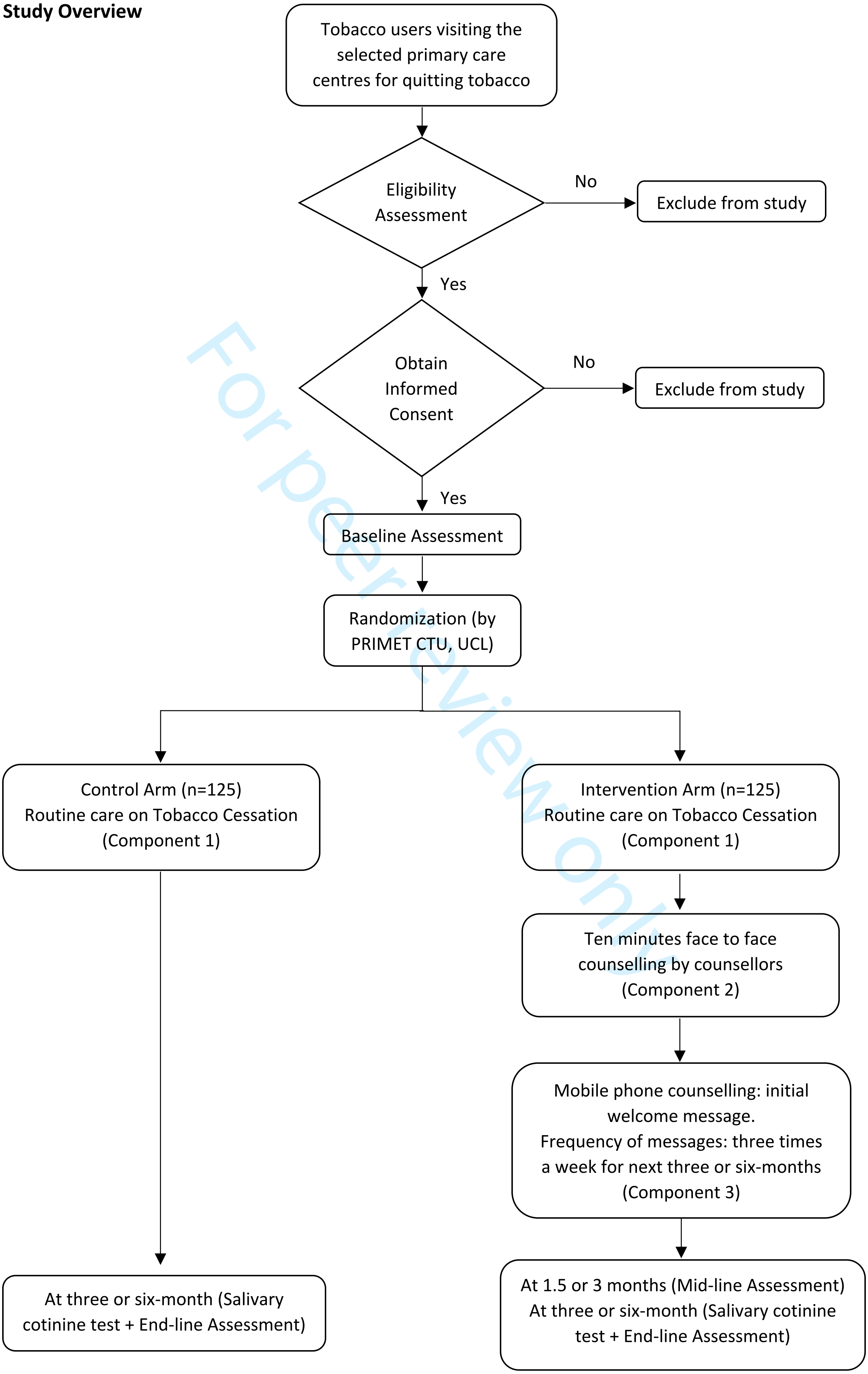

For peer review only - http://bmjopen.bmj.com/site/about/guidelines.xhtml

SPIRIT
STANDARD PROTOCOL ITEMS: RECOMMENDATIONS FOR INTERVENTIONAL TRIALS

SPIRIT 2013 Checklist: Recommended items to address in a clinical trial protocol and related documents*

Section/item	ItemNo	Description	Yes/No	PageNo
**Administrative information**				
Title	1	Descriptive title identifying the study design, population, interventions, and, if applicable, trial acronym	Yes	1
Trial registration	2a	Trial identifier and registry name. If not yet registered, name of intended registry	Yes	2
	2b	All items from the World Health Organization Trial Registration Data Set	Yes	2
Protocol version	3	Date and version identifier	Yes	1-13
Funding	4	Sources and types of financial, material, and other support	Yes	10
Roles and responsibilities	5a	Names, affiliations, and roles of protocol contributors	Yes	1
	5b	Name and contact information for the trial sponsor	Yes	10
	5c	Role of study sponsor and funders, if any, in study design; collection, management, analysis, and interpretation of data; writing of the report; and the decision to submit the report for publication, including whether they will have ultimate authority over any of these activities	N/A	N/A
	5d	Composition, roles, and responsibilities of the coordinating centre, steering committee, endpoint adjudication committee, data management team, and other individuals or groups overseeing the trial, if applicable (see Item 21a for data monitoring committee)	N/A	N/A

For peer review only

**Introduction**				
Background and rationale	6a	Description of research question and justification for undertaking the trial, including summary of relevant studies (published and unpublished) examining benefits and harms for each intervention	Yes	3-4
	6b	Explanation for choice of comparators	Yes	4
Objectives	7	Specific objectives or hypotheses	Yes	4
Trial design	8	Description of trial design including type of trial (eg, parallel group, crossover, factorial, single group), allocation ratio, and framework (eg, superiority, equivalence, noninferiority, exploratory)	Yes	4
**Methods: Participants, interventions, and outcomes**				
Study setting	9	Description of study settings (eg, community clinic, academic hospital) and list of countries where data will be collected. Reference to where list of study sites can be obtained	Yes	4
Eligibility criteria	10	Inclusion and exclusion criteria for participants. If applicable, eligibility criteria for study centres and individuals who will perform the interventions (eg, surgeons, psychotherapists)	Yes	6
Interventions	11a	Interventions for each group with sufficient detail to allow replication, including how and when they will be administered	Yes	6-7
	11b	Criteria for discontinuing or modifying allocated interventions for a given trial participant (eg, drug dose change in response to harms, participant request, or improving/worsening disease)	N/A	N/A

For peer review only - http://bmjopen.bmj.com/site/about/guidelines.xhtml

	11c	Strategies to improve adherence to intervention protocols, and any procedures for monitoring adherence (eg, drug tablet return, laboratory tests)	N/A	N/A
	11d	Relevant concomitant care and interventions that are permitted or prohibited during the trial	N/A	N/A
Outcomes	12	Primary, secondary, and other outcomes, including the specific measurement variable (eg, systolic blood pressure), analysis metric (eg, change from baseline, final value, time to event), method of aggregation (eg, median, proportion), and time point for each outcome. Explanation of the clinical relevance of chosen efficacy and harm outcomes is strongly recommended	Yes	8
Participant timeline	13	Time schedule of enrolment, interventions (including any run-ins and washouts), assessments, and visits for participants. A schematic diagram is highly recommended (see Figure)	Yes	7&13
Sample size	14	Estimated number of participants needed to achieve study objectives and how it was determined, including clinical and statistical assumptions supporting any sample size calculations	Yes	8
Recruitment	15	Strategies for achieving adequate participant enrolment to reach target sample size	Yes	6
**Methods: Assignment of interventions (for controlled trials)**				
Allocation:				
Sequence generation	16a	Method of generating the allocation sequence (eg, computer-generated random numbers), and list of any factors for stratification. To reduce predictability of a random sequence, details of any planned restriction (eg, blocking) should be provided in a separate document that is unavailable to those who enrol participants or assign interventions	Yes	6

Allocation concealment mechanism	16b	Mechanism of implementing the allocation sequence (eg, central telephone; sequentially numbered, opaque, sealed envelopes), describing any steps to conceal the sequence until interventions are assigned	Yes	6
Implementation	16c	Who will generate the allocation sequence, who will enrol participants, and who will assign participants to interventions	Yes	6
Blinding (masking)	17a	Who will be blinded after assignment to interventions (eg, trial participants, care providers, outcome assessors, data analysts), and how	Yes	6
	17b	If blinded, circumstances under which unblinding is permissible, and procedure for revealing a participant's allocated intervention during the trial	N/A	N/A
**Methods: Data collection, management, and analysis**				
Data collection methods	18a	Plans for assessment and collection of outcome, baseline, and other trial data, including any related processes to promote data quality (eg, duplicate measurements, training of assessors) and a description of study instruments (eg, questionnaires, laboratory tests) along with their reliability and validity, if known. Reference to where data collection forms can be found, if not in the protocol	Yes	7
	18b	Plans to promote participant retention and complete follow-up, including list of any outcome data to be collected for participants who discontinue or deviate from intervention protocols	Yes	7
Data management	19	Plans for data entry, coding, security, and storage, including any related processes to promote data quality (eg, double data entry; range checks for data values). Reference to where details of data management procedures can be found, if not in the protocol	Yes	8
Statistical methods	20a	Statistical methods for analysing primary and secondary outcomes. Reference to where other details of the statistical analysis plan can be found, if not in the protocol	Yes	8
	20b	Methods for any additional analyses (eg, subgroup and adjusted analyses)	Yes	8

For peer review only - http://bmjopen.bmj.com/site/about/guidelines.xhtml

	20c	Definition of analysis population relating to protocol non-adherence (eg, as randomised analysis), and any statistical methods to handle missing data (eg, multiple imputation)	Yes	8
**Methods: Monitoring**				
Data monitoring	21a	Composition of data monitoring committee (DMC); summary of its role and reporting structure; statement of whether it is independent from the sponsor and competing interests; and reference to where further details about its charter can be found, if not in the protocol. Alternatively, an explanation of why a DMC is not needed	N/A	N/A
	21b	Description of any interim analyses and stopping guidelines, including who will have access to these interim results and make the final decision to terminate the trial	N/A	N/A
Harms	22	Plans for collecting, assessing, reporting, and managing solicited and spontaneously reported adverse events and other unintended effects of trial interventions or trial conduct	Yes	7
Auditing	23	Frequency and procedures for auditing trial conduct, if any, and whether the process will be independent from investigators and the sponsor	N/A	N/A
**Ethics and dissemination**				
Research ethics approval	24	Plans for seeking research ethics committee/institutional review board (REC/IRB) approval	Yes	4-5
Protocol amendments	25	Plans for communicating important protocol modifications (eg, changes to eligibility criteria, outcomes, analyses) to relevant parties (eg, investigators, REC/IRBs, trial participants, trial registries, journals, regulators)	N/A	N/A
Consent or assent	26a	Who will obtain informed consent or assent from potential trial participants or authorised surrogates, and how (see Item 32)	Yes	4-5
	26b	Additional consent provisions for collection and use of participant data and biological specimens in ancillary studies, if applicable	N/A	N/A

For peer review only - http://bmjopen.bmj.com/site/about/guidelines.xhtml

Confidentiality	27	How personal information about potential and enrolled participants will be collected, shared, and maintained in order to protect confidentiality before, during, and after the trial	Yes	4-5
Declaration of interests	28	Financial and other competing interests for principal investigators for the overall trial and each study site	Yes	10
Access to data	29	Statement of who will have access to the final trial dataset, and disclosure of contractual agreements that limit such access for investigators	Yes	10
Ancillary and post-trial care	30	Provisions, if any, for ancillary and post-trial care, and for compensation to those who suffer harm from trial participation	N/A	N/A
Dissemination policy	31a	Plans for investigators and sponsor to communicate trial results to participants, healthcare professionals, the public, and other relevant groups (eg, via publication, reporting in results databases, or other data sharing arrangements), including any publication restrictions	Yes	4
	31b	Authorship eligibility guidelines and any intended use of professional writers	Yes	9
	31c	Plans, if any, for granting public access to the full protocol, participant-level dataset, and statistical code	N/A	N/A
**Appendices**				
Informed consent materials	32	Model consent form and other related documentation given to participants and authorised surrogates	Yes	4-5
Biological specimens	33	Plans for collection, laboratory evaluation, and storage of biological specimens for genetic or molecular analysis in the current trial and for future use in ancillary studies, if applicable	N/A	N/A

*It is strongly recommended that this checklist be read in conjunction with the SPIRIT 2013 Explanation & Elaboration for important clarification on the items. Amendments to the protocol should be tracked and dated. The SPIRIT checklist is copyrighted by the SPIRIT Group under the Creative Commons "Attribution-NonCommercial-NoDerivs 3.0 Unported" license.

For peer review only - http://bmjopen.bmj.com/site/about/guidelines.xhtml

BMJ Open

Exploratory randomized trial of face to face and mobile phone counselling against usual care for tobacco cessation in Indian primary care: a randomized controlled trial protocol for project CERTAIN.

Journal:	*BMJ Open*
Manuscript ID	bmjopen-2021-048628.R1
Article Type:	Protocol
Date Submitted by the Author:	29-Jul-2021
Complete List of Authors:	Panda, Rajmohan; Public Health Foundation of India, research<br>Omar, Rumana; University College London,<br>Hunter, Rachael; University College London, Research Dept of Primary Care and Population Health<br>Prabhu, Rajath R; Public Health Foundation of India, research<br>Mishra, Arti; Independent Consultant, Research<br>Nazareth, Irwin; UCL, Primary Care and Population Health
**Primary Subject Heading**:	Public health
Secondary Subject Heading:	Epidemiology, Smoking and tobacco
Keywords:	Protocols & guidelines < HEALTH SERVICES ADMINISTRATION & MANAGEMENT, PRIMARY CARE, QUALITATIVE RESEARCH, PUBLIC HEALTH

SCHOLARONE™
Manuscripts

Running Title: Counselling intErvention foR smokeless Tobacco cessAtion in INdian primary care: The CERTAIN study

Title: Exploratory randomized trial of face to face and mobile phone counselling against usual care for tobacco cessation in Indian primary care: a randomized controlled trial protocol for project CERTAIN.

Correspondence to: Dr Rajmohan Panda, raj.panda@phfi.org, Public Health Foundation of India, Plot Number 47, Sector 44, Gurugram, Haryana, India

Rajmohan Panda, Research Division, Public Health Foundation of India, Plot Number 47, Sector 44, Gurugram, Haryana, India

Rumana Omar, Department of Statistical Science, University College London, Gower Street, London, WC1E 6BT, United Kingdom

Rachael Hunter, Department of Primary Care and Population Sciences, University College London, Gower Street, London, WC1E 6BT, United Kingdom

Rajath R Prabhu, Research Division, Public Health Foundation of India, Plot Number 47, Sector 44, Gurugram, Haryana, India

Arti Mishra, Research, Independent Consultant

Irwin Nazareth, Department of Primary Care and Population Sciences, University College London, Gower Street, London, WC1E 6BT, United Kingdom

Word Count: 3395 (excluding title page, abstract, references, figures and tables)

Keywords/Phrases: tobacco cessation; smokeless tobacco; mobile counselling; primary care; formative research; exploratory randomized controlled trial

For peer review only

For peer review only - http://bmjopen.bmj.com/site/about/guidelines.xhtml

Abstract

Introduction: Despite widespread use of smokeless tobacco products by people in within the Indian sub-continent, there is little awareness amongst Indians of its health hazards when compared to smoked tobacco. We hypothesize that mobile phone counselling will be feasible, and effective for smokeless tobacco cessation intervention in India. This paper presents the protocol of the development and conduct of an exploratory trial before progression to a full randomized controlled trial.

Methods and Analysis: An exploratory randomized controlled trial will be conducted in urban primary health centres in the state of Odisha, India. A total of 250 smokeless tobacco users will be recruited to the study (125 in each arm). Participants in the intervention arm will receive routine care together with a face-to-face counselling intervention followed by advice and reminder mobile messages. The control arm will receive routine care, delivered by a primary care physician based on 'Ask' and 'Advice'. All participants will be followed up for three months from the first counselling session. The primary outcome of this trial is to assess the feasibility to carry out a full randomized controlled trial.

Ethics and Dissemination: Ethical approvals were obtained from the Institutional Ethics Committee of Public Health Foundation of India, Health Ministry's Screening Committee – ICMR, Odisha State Ethics Board, and also from University College London Research Ethics Committee, UK. The study findings will be published in a peer-reviewed scientific journal.

Trial Registration Number: CTRI Registration Number CTRI/2019/05/019484; dated May 31, 2019 (http://ctri.nic.in/Clinicaltrials/pmaindet2.php?trialid=30948&EncHid=&userName=Tobacco%20cessation)

Article Summary:

Strengths and limitations of this study:

- Evidence on the effectiveness of smokeless tobacco cessation intervention in primary care is scarce. The results of this study will inform on the feasibility of conducting an effectiveness trial in the future.
- Randomization with allocation concealment, blinded assessment of outcome measures, and registration of the protocol in the trial registry will minimize potential bias.
- Mobile phone technology will be used to deliver intervention among the participants.
- Providing training to health professionals will lead to an improved understanding of the delivery of low-cost tobacco cessation interventions in primary care.
- The study findings cannot be generalized to other healthcare settings as this study is limited to Indian urban health settings (i.e., Urban Public Health Centres).

For peer review only - http://bmjopen.bmj.com/site/about/guidelines.xhtml

Introduction

Tobacco use is responsible for almost eight million deaths each year or a death every six seconds.[1,2] About 80% of 1.3 billion tobacco consumers across the globe live in Low-and Middle-Income Countries (LMICs).[1] In LMICs like India, the tobacco problem is complex as the country has a diverse population with a mixture of cultures, religions, and practices. The majority of the tobacco users in India use a variety of tobacco products – combustible, non-combustible, or both. As per the Global Adult Tobacco Survey 2 (GATS 2), 28.6% of the adult population in India consumes tobacco (10.7% of smoking and 21.4% of smokeless) making it the second-largest consumer in the world.[3]

Widespread use of smokeless tobacco (SLT) products occurs in countries such as in India and Bangladesh accounting for 232 of 248 million SLT users worldwide (i.e. from 21 countries).[4] SLT products such as "*tambaaku*", "*gurkha*", "*zarda*", "*khaini*", "*mawa*" and "*pan masala*" are widely used in many states in India as SLT consumption is culturally accepted and is considered common practice.[5] In India, SLT use is double that of combustible tobacco, making it the single most common form of tobacco used by men and women (29.6% and 12.8% respectively).[5] According to GATS 2 survey, the state of Odisha was one where the largest proportion of adults consumed SLT products (43%) compared with the national average (21.4%).[6] Despite strong evidence of SLT[7] on cancer of the oral cavity, pharynx, oesophagus, and also responsible for a large proportion of tobacco-related cancers in India, there is a misconception that SLT is relatively harmless .[8]

Primary care health professionals, the first contact for patients accessing help, play a key role in the health education of the hazards of tobacco. They are best placed to counsel against tobacco use and promote cessation. Studies on brief interventions by healthcare providers are effective in motivating tobacco users to quit tobacco.[9,10] A systematic review on SLT cessation intervention from both high income countries (HICs) and LMICs showed that behavioural interventions are effective in achieving cessation.[11] However, the overburdened primary care physicians in India have limited time for counselling[2,12,13] and it is also difficult to get users to attend follow-up visits as journeys to primary care clinics would incur a loss of income.[12]

In India, mobile phones are widely used in both rural and urban settings. India with 1173.7 million (98.2%) mobile phone users is the second-largest user base in the world.[14] Mobile phone subscribers in India increased from 1187 million in June 2019 to 1195.2 million by September 2019.[14] A meta-analysis of 104 studies including randomized trials and quasi-randomized trials on the effectiveness of telephone counselling for smoking tobacco cessation showed significant but modest effect sizes in HICs.[15] Effectiveness increases with treatment intensity.[16] A review of 26 trials on smoking cessation demonstrated that automated text messaging interventions were more effective than minimal smoking cessation support (RR 1.54, 95% CI 1.19 to 2.00; I2 = 71%; 13 studies) and text messaging added to other smoking cessation interventions was more effective than other individual smoking cessation interventions (RR 1.59, 95% CI 1.09 to 2.33; I2 = 0%, 4 studies).[17] Another meta-analysis of 13 trials reported smoking quit rates with text messaging intervention were 35% higher than quit rates for controls (OR = 1.35, 95% CI = 1.23, 1.49).[18] Other reviews of studies from HICs suggest that such interventions can increase the chance of quitting smoking tobacco from 39% to 80%.[19,20] Studies on SLT cessation that included randomized controlled trials conducted in HICs as well as some LMICs showed that behavioural cessation interventions led to quit rates between 9% to 51.5% at six months.[11,21] There are however little data on the efficacy and effectiveness of such intervention combined with mobile phone-based counselling in LMIC like India. Improved participation in tobacco cessation programmes through the use of mobile phone technology can have a substantial impact on a population at risk even with a small effect size.[22]

For peer review only - http://bmjopen.bmj.com/site/about/guidelines.xhtml

There is an urgent need to test the effectiveness of the use of mobile phone messages for SLT cessation in the Indian primary care setting.[23] We hypothesize that a face-to-face intervention offered by a tobacco counsellor together with low-cost mobile-based counselling has the potential to deliver a high-quality cessation intervention to SLT users. Prior to that we need to test the feasibility and acceptability of recruitment, delivery of the intervention and the follow-up of participants within a randomized controlled trial.

Aim and Objectives

Our overarching future aim is to evaluate within a randomized controlled trial, the clinical effectiveness (as measured by tobacco cessation) and cost-effectiveness of a complex intervention of face-to-face counselling coupled with mobile phone messaging delivered to SLT users visiting Indian primary care clinics in addition to routine care provided by the primary care professional against routine care alone. Prior to undertaking a full trial, we aim to develop and finalise the intervention and then test its acceptability and feasibility. Specific objectives of this study are to assess within an exploratory randomized controlled trial, the proportion of:

1. all primary care smokeless tobacco user attendees approached to take part in the trial that consent to randomization.
2. those who are randomized to the intervention, that comply with the intervention.
3. those who are randomized on whom the follow-up data can be collected.
4. missing data on all research measures administered at baseline and three months that will be used to assess the primary and secondary outcomes for conducting effectiveness randomized controlled trial in future.

Methodology

Patient and Public Involvement

The patients and public were involved in the formulation of the study hypothesis from previous research studies.[24,25] The research questions, design and outcome measures were derived from previous interaction with different stakeholders including patient and public. Tobacco users and other stakeholders (including primary care physicians, counsellors) will be involved in the development of two components of the intervention i.e., face-to-face counselling and mobile phone messages. These will be done by in-depth interviews and focus group discussion in the formative phase of the study *(Appendix A)*. The summary of the results of the study will be made available to patients and public at the end of the project period.

Study Design

The study will be conducted in two different phases. The first phase is a formative phase *(Appendix A)* and the second phase is an exploratory, parallel-group, randomized controlled trial, with pre-and post-test assessments. The duration of the study will be 18 months. The first phase will last for 8 months whereas the second phase will occur over 10 months (see table 1). The study flowchart is provided in figure 1.

Study Setting

The study will be conducted in urban primary health centres (UPHCs). All UPHCs in Berhampur city of Ganjam district of Odisha will be selected for this study.

Study Population

The study participants will include adult (above 18 years) SLT users who visit UPHCs in Berhampur city, Odisha. Participants must be current SLT users (i.e., users in the last three months), aged 18 years and

For peer review only - http://bmjopen.bmj.com/site/about/guidelines.xhtml

above, have a mobile phone with a valid contact number, and willing to consent to participate in the study.

Participants below the age of 18 years, who do not have the mental capacity to consent, with illness limiting their adherence and follow-up within the study, will be excluded.

Interventions

The intervention arm includes routine care (component 1) along with a single ten-minute face-to-face intervention based on 5A's approach (component 2), followed by mobile phone counselling (component 3). The control arm will be the provision of routine care alone (i.e. component 1).

CERTAIN Intervention: The intervention will be a combination of the following components -

1) Component one or delivery of "routine care" as delivered by a primary care physician over one to two minutes. This component will be delivered to both the intervention and control arms of the study. This is based on 'Ask' and 'Advice'. Under 'Ask', the physician will ask about consumption of SLT and under 'Advice', the physician will advise participants about the benefits of complete abstinence in a clear personalized manner.

2) Component two will include a single ten-minute face-to-face intervention delivered by a practice-based counsellor. This component will include brief standardized advice provided to participants based on 5A's approach[26] to tobacco cessation. Under this approach the counsellor will 'Ask' the participants if they use SLT; 'Advice' them on the importance of quitting (following the same script of component 1). Additionally, they will 'Assess' their willingness to 'quit now'; offer them 'Assistance' in the form of Nicotine Replacement Therapy (NRT) and/or provide them with a referral for behavioural support; and 'Arrange' a follow-up to check on progress. This will conclude with their enrolment to receive Component three – mobile phone intervention.

3) Component three will also follow 5A's approach to tobacco cessation. The intervention will include an initial message after 48 hours of recruitment, to remind them to quit tobacco followed by messages occurring three times a week for the next three months. Each follow-up message will offer behavioural messages to support quitting; information about health risks of tobacco use and benefits of quitting, and advice on withdrawal symptoms and coping strategies.

Training of tobacco cessation counsellors delivering the intervention: Counsellors at UPHCs are allied healthcare workers and will be trained by the members of the research team to deliver counselling intervention. They will not be involved in data collection or data analysis. Their training will be done using a standardized training manual (hardcopy of study manual will be provided to these counsellors) to maintain the fidelity of the intervention.

Outcomes

The main outcomes expected from this exploratory trial include:

1. The proportion of all the tobacco users approached who consent to randomization;
2. The proportion of those randomized who comply with the intervention; and
3. The proportion of those randomized on whom follow-up data are collected at three months;

We will also assess the feasibility of assessing outcomes of primary interest in the main trial as listed below:

For peer review only - http://bmjopen.bmj.com/site/about/guidelines.xhtml

- "Self-reported tobacco abstinence" of seven days confirmed by a salivary cotinine test.
- Self-reported motivation and intention to quit.
- Cost-effectiveness as cost per disability-adjusted life year (DALY) averted.
- Cost-effectiveness as cost per quality-adjusted life year (QALY) gained.

The outcomes will be assessed at baseline on recruitment to the study and the end of three months. At baseline, socio-demographic details such as age, gender, educational level, marital status, religion, community, the economic status will be recorded. Variables related to tobacco consumption will be recorded including, the number of SLT products per day (frequency), age of initiation, number of previous cessation attempts, and longest period of cessation. The data on quit attempts, the status of tobacco use, and challenges will be recorded during baseline and end-point. The biochemical validation of self-reported tobacco abstinence for seven days will be assessed using salivary cotinine at the end of three months. The rapid test kit works through visual interpretation of colour developed on the kit and is instant in processing.[27,28] Participants will be approached by the trial team members to provide a small sample of saliva which will be collected through passive drooling. Collection, transportation, and disposition of the biological samples and the kit will be managed at the study site by the site coordinator and research team. Cotinine is a preferred marker for tobacco use because cotinine stays in the body much longer than nicotine. The presence of cotinine in saliva or urine is a reliable indicator of tobacco use. Cotinine in vivo has a half-life of about twenty hours and lasts for about 3-4 days in the saliva and urine.[29,30]

Sample Size

Based on the primary outcomes in this exploratory trial of recruitment to the study and attrition, a total of 250 participants (125 participants per group) is required to estimate an anticipated proportion of 50% recruitment of participants with a 95% confidence interval (CI) of 44% to 57% and 20% attrition at follow-up with a 95% CI of 15% to 25%. The sample size was calculated based on estimating proportions with a specified level of precision at the 95% level as measured by the width of the confidence interval using the Sample Size Tables for Clinical Studies software.[31]

Randomization and Allocation

Randomization will be done at the level of individual eligible participants who have provided informed consent. Randomization will be stratified by study practice site (UPHCs) using random permuted blocks of varying block sizes from 4 to 10, and a 1:1 allocation to intervention or control arm. A randomization list will be prepared by an independent statistician (from PRIMENT CTU, UCL) not involved with this study. This list will be mailed to an independent staff member at the study site in India who will be responsible for the allocation of participants to the respective intervention arm. The independent staff member will assign a unique identification number to each of the consented participants and will maintain a list of those allocated to the respective study arm. The updated list will then be sent to the randomization coordinator who will be responsible for overseeing the recruitment and randomization process. The randomization coordinator will remove all the participant identifiers from the list and finally send the list to the statistician for the purpose of analysis.

The trial will ensure a clear separation between staff that collect outcome data and those that deliver the intervention. Staff collecting outcome data will be blinded to the group assignment. None of the intervention staff, primary care physicians, and behavioural counsellors will collect outcome data. All investigators, staff, and participants will be kept masked to outcome measurements and any preliminary results emerging from the trial.

For peer review only - http://bmjopen.bmj.com/site/about/guidelines.xhtml

Data Management

Baseline and end-point data will be collected from participants at UPHC using surveys in paper format. This will be entered electronically in the database developed in MS Access (Microsoft Office 2019). The quality of the data will be monitored by a data supervisor for completeness, validity, and integrity. In case of incomplete data or inconsistent responses, the data supervisor will verify these with the study participants and update the entries in the database. The database will incorporate a range of inconsistency checks to limit data entry errors. Time-stamped audit trail will also be built in the database to independently record the date and time of operator entries and actions that create, modify, or delete electronic records.

Data Analysis

Statistical Analysis: Participant characteristics will be summarised using mean and standard deviation [SD] or median and interquartile range for continuous variables, and number & percentages for the categorical variables. The proportion of participants recruited and lost to follow-up at the end-point will be estimated with 95% confidence intervals. As part of the secondary analyses, regression models will be used to estimate the intervention effects with 95% confidence intervals for a number of pre-specified outcomes (i.e. tobacco abstinence, self-reported motivation and intention to quit) after adjusting for baseline values. The choice of the regression model (for example, linear, logistic etc) will depend on the type of the outcome (for example, continuous, binary, count etc.). These analyses will be done out on an intention to treat (as randomized) basis and multiple imputation will be used to handle missing outcome values if considered appropriate. The extent of missing data for each variable and the percentage of participants adhering to the intervention will be reported. Attrition levels by randomized group and the characteristics of participants who are lost to follow-up will also be reported. All analyses will be done using STATA software version 16 (StataCorp. 2019. *Stata Statistical Software: Release 16*. College Station, TX: StataCorp LLC) or updated versions. A detailed statistical analysis plan will be drawn up nearer the analysis stage, prior to the database lock.

Economic Evaluation: The aim will be to evaluate the feasibility of calculating the cost-effectiveness of routine care along with intervention compared to routine care alone from an Indian health care cost perspective over three months. This will include the level of data completeness of self-completed questionnaires of tobacco-related health care resource use, estimation of the intervention, and routine care costs. The feasibility of calculating cost per DALY averted, particularly in relation to potential cases of cancer avoided, as part of the main trial will also be evaluated in addition to data requirements for a decision model to evaluate the lifetime cost-effectiveness of the intervention calculated as the cost per QALY gained.

Trial Status

The two major COVID-19 pandemic waves have affected India (wave one in March 2020 and wave two in April 2021). This has delayed the recruitment of the trial since out-patient departments (OPDs) were not operational or operating with limited staff. Our current plan is to start recruitment by mid-July 2021. As per the original proposal, the follow-up was for a period of six months, our current plan for follow-up is now for three months.

Discussion

To the best of our knowledge, this is the first study evaluating a complex low-cost intervention of two components i.e., face-to-face counselling along with mobile phone messaging counselling delivered to SLT users in low resource Indian settings. The first component of the intervention is based on 5A's approach which is known to be effective.[32] However, such a brief intervention may have a limited

For peer review only - http://bmjopen.bmj.com/site/about/guidelines.xhtml

effect if the person is not ready to quit on account of other factors associated with difficulty in quitting tobacco.[33] However, follow-up quitting advice to tobacco users can increase quit attempts and quit rates[32] and this will be achieved through, the mobile phone messaging. Mobile based interventions are widely used in the developed world and research on smoking cessation suggests that they can increase tobacco quitting by 25-50%.[20] A study conducted in Sweden assessed the heterogeneous treatment effects of text messaging intervention among smokers and found that the effect was less pronounced among the participants with stronger nicotine dependence.[34] Tailoring of such interventions for specific individuals needs to be considered, as each individual will need messaging according to their beliefs and their readiness to quit. The mobile messages in our study will be developed with this context in mind. To date, there have been no evaluations conducted to assess the effectiveness of such complex low-cost intervention in low resource settings and primary care. The development and delivery of comprehensive, tailored primary care behavioural and mobile phone health technology interventions will enhance the understanding of low-cost long-term prevention intervention for tobacco cessation. Recent evidence has emerged on the association of tobacco use with novel coronavirus disease.[35,36] The use of SLT often involves hand-to-mouth contact that can promote the spread of COVID-19. Another behaviour that can spread infections associated with the use of SLT is the spitting of or projection of excess saliva produced during the chewing process.[37] Whilst, the struggle with coronavirus infection control continues, we have an opportunity to expand cessation intervention specifically designed for SLT use and promote good infection control practice.

Strengths and Limitations

This study has many strengths. The results of this study will inform the feasibility of conducting effectiveness trials in the future. Incorporating messaging technology in routine clinical practice will be time-efficient as the messages can be delivered anywhere, at fixed times, and directly to the participant with negligible direct contact whilst also ensuring privacy. The impact of this research on the training of health professionals will lead to an improved understanding of the delivery of low-cost tobacco cessation interventions in primary care. There are however some limitations to the study. As the study is being conducted in urban health centres in Berhampur city, Odisha, the findings would not be generalisable to other healthcare settings as well as to the whole population of the Odisha state.

Research Impact

In this study, SLT cessation intervention will be developed using an innovative method including a formative research phase, where the SLT cessation intervention will be developed; and the exploratory randomized controlled trial phase, where the evaluation of this intervention will be conducted. The evidence from this exploratory study will inform the acceptability and feasibility of SLT cessation interventions in users of SLT and will inform the conduct of a larger multi-centric trial across several centres and countries. Findings from this study will provide insights for designing similar studies and appropriate interventions in tobacco cessation and non-communicable diseases. Since research in this area is in its infancy in LMIC, this work will provide an impetus for researchers working on tobacco cessation to generate new evidence and allow them to adapt the tools developed and piloted in this study in real time practice.

Contributions: All authors contributed to the manuscript. RP (PI of the study – India) and IN (PI of the study – UK) designed the study, led on writing, review and finalising the paper. RO provided statistical guidance. RH provided inputs on designing the economic evaluation section of the study. AM provided inputs on designing the formative research section of the study. RRP reviewed and assisted in writing

For peer review only - http://bmjopen.bmj.com/site/about/guidelines.xhtml

the early drafts of the paper. All authors provided critical feedback and helped in shaping the manuscript.

Funding statement: This research work is funded by the Joint Global Health Trials Initiative MRC/DfID/Wellcome Trust (ref: MR/P021166/1)

Sponsor: This research work is sponsored by University College London, Gower Street, London, WC1E 6BT, United Kingdom

Competing interests: None declared

Ethics and Dissemination: Ethics approval has been obtained from the Institutional Ethical Committee at Public Health Foundation of India (PHFI) (ref: TRC-IEC-391/19; dated May 29, 2019). At the national level, ethical clearance has also been obtained from the Health Ministry's Screening Committee (HMSC), led by the Indian Council of Medical Research (ICMR) (ref: 2019-3581; dated December 11, 2019). Also, local level approval was obtained from the Odisha State Ethics Board (ref: 191/PMU/187/17; dated November 14, 2019). In the UK, ethical clearance was obtained by the UCL Research Ethics Committee (ref: 5686/001, dated October 1, 2019). The study has been registered at Clinical Trials Registry India (reference number CTRI/2019/05/019484). All the participants (participating in both the phases in this study) will be provided with a participant information sheet (PIS), providing details of the study. Following this, voluntary written consent for participation will also be taken from them *(Appendix B)*. All the data that will be collected for the study will be stripped of any personal identifiers and the data will be stored in PHFI's data repository. The data will only be accessible to the principal investigator and trial team analysing the data. To ensure confidentiality, data shared to project team members will be blinded for any identifying participant information. The findings of the study will be published in peer-reviewed journals.

Data sharing statement: The authors will make available data underpinning research publications at the time of such publication, or at any time prior to publication when reasonably requested, as well as any original software that may be required to view such data.

References

1. World Health Organization. Tobacco [Internet]. [cited 2020 Jul 20]Who.int. World Health Organization: WHO; 2018. Available from: https://www.who.int/news-room/fact-sheets/detail/tobacco

2. Amit S, Bhambal A, Saxena V et al. Tobacco cessation and counseling: a dentists' perspective in Bhopal city, Madhya Pradesh. Indian J Dent Res Off Publ Indian Soc Dent Res. 2011 Jun;22(3):400–3.

3. GATS 2 Global Adult Tobacco Survey [Internet]. [cited 2020 Jul 20] Available from: https://www.who.int/tobacco/surveillance/survey/gats/GATS_India_2016-17_FactSheet.pdf

4. GATS Atlas Online [Internet]. gatsatlas.org. [cited 2020 Jul 20]. Available from: http://gatsatlas.org/pdf/mobile/index.html#p=38

5. Murthy P, Subodh BN, Sinha D, et al. Smokeless tobacco (SLT) use and cessation in India: Lessons from user and health care provider perspectives. Asian J Psychiatry. 2018 Feb;32:137–42.

6. GATS 2 Global Adult Tobacco Survey [Internet]. [cited 2020 Jul 20]. Available from: https://tiss.edu/uploads/files/Odisha.pdf

For peer review only - http://bmjopen.bmj.com/site/about/guidelines.xhtml

7. Monika S, Dineshkumar T, Priyadharini S, et al. Smokeless Tobacco Products (STPs) Harbour Bacterial Populations with Potential for Oral Carcinogenicity. Asian Pac J Cancer Prev. 2020 Mar 1;21(3):815–24.

8. Raute LJ, Sansone G, Pednekar MS, et al. Knowledge of health effects and intentions to quit among smokeless tobacco users in India: findings from the International Tobacco Control Policy Evaluation (ITC) India Pilot Survey. Asian Pac J Cancer Prev APJCP. 2011;12(5):1233–8.

9. Gordon JS, Andrews JA, Crews KM, et al. The 5A's vs 3A's plus proactive quitline referral in private practice dental offices: preliminary results. Tob Control. 2007 Aug;16(4):285–8.

10. Gordon JS, Andrews JA, Albert DA, et al. Tobacco Cessation via Public Dental Clinics: Results of a Randomized Trial. Am J Public Health. 2010 Jul;100(7):1307–12.

11. Nethan ST, Sinha DN, Chandan K, et al. Smokeless tobacco cessation interventions: A systematic review. Indian J Med Res. 2018 Apr 1;148(4):396.

12. Champassak SL, Goggin K, Finocchario-Kessler S, et al. A qualitative assessment of provider perspectives on smoking cessation counselling. Journal of Evaluation in Clinical Practice. 2014 Jun 1;20(3):281–287.

13. Jradi H. Awareness, practices, and barriers regarding smoking cessation treatment among physicians in Saudi Arabia. J Addict Dis. 2017 Jan 2;36(1):53–9.

14. Bhawan M. The Indian Telecom Services Performance Indicators, July – September, 2019 [Internet]. [cited 2020 Jul 20] 2020 Jan. Available from: https://trai.gov.in/sites/default/files/PIR_08012020_0.pdf

15. Matkin W, Ordóñez-Mena JM, Hartmann-Boyce J. Telephone counselling for smoking cessation. Cochrane Database Syst Rev. 2019 May 02 [Internet].[cited 2020 May 24];(5). Available from: https://www.cochranelibrary.com/cdsr/doi/10.1002/14651858.CD002850.pub4/full

16. Fiore M, United States. Tobacco Use And Dependence Guideline Panel. Treating tobacco use and dependence : 2008 update. Darby, Pa.: Diane Pub. Co; 2009.

17. Whittaker R, McRobbie H, Bullen C, et al. Mobile phone text messaging and app-based interventions for smoking cessation. Cochrane Database Syst Rev. 2019 Oct 22;10:CD006611

18. Spohr SA, Nandy R, Gandhiraj D, et al. Efficacy of SMS Text Message Interventions for Smoking Cessation: A Meta-Analysis. J Subst Abuse Treat. 2015 Sep;56:1–10.

19. Whitehouse E, Lai J, Golub JE, et al. A systematic review of the effectiveness of smoking cessation interventions among patients with tuberculosis. Public Health Action. 2018 Jun 21;8(2):37–49.

20. Stead LF, Hartmann-Boyce J, Perera R, et al. Telephone counselling for smoking cessation. Cochrane Database Syst Rev. 2013 Aug 12;(8):CD002850.

21. Messina J, Freeman C, Rees A, et al. Smokeless tobacco: South Asians -Reviews 1 and 2 - Effectiveness and contextual factors Systematic review of effectiveness of smokeless tobacco interventions for South Asians and a review of contextual factors relating to smokeless tobacco among South Asian users and health care providers [Internet]. [cited 2020 Jul 20]. Available from: https://www.nice.org.uk/guidance/ph39/documents/smokeless-tobacco-cessation-south-asians-evidence-reviews-1-and-22

For peer review only

For peer review only - http://bmjopen.bmj.com/site/about/guidelines.xhtml

22. Baskerville NB, Struik LL, Hammond D, et al. Effect of a mobile phone intervention on quitting smoking in a young adult population of smokers: randomized controlled trial study protocol. JMIR Res Protoc. 2015 Jan 19;4(1):e10.

23. IndianJMedRes1406707-3538149_094941.pdf [Internet]. [cited 2020 Jul 20]. Available from: http://www.ijmr.org.in/temp/IndianJMedRes1406707-3538149_094941.pdf

24. Panda R, Persai D, Venkatesan S. Missed opportunities for brief intervention in tobacco control in primary care: patients' perspectives from primary health care settings in India. BMC Health Services Research. 2015 Feb 1;15(1).

25. Panda R, Persai D, Venkatesan S, Ahluwalia JS. Physician and patient concordance of report of tobacco cessation intervention in primary care in India. BMC Public Health. 2015 May 2;15(1).

26. Tobacco Use and Dependence Guideline Panel. Treating Tobacco Use and Dependence: 2008 Update [Internet]. Nih.gov. US Department of Health and Human Services; 2008. Available from: https://www.ncbi.nlm.nih.gov/books/NBK63952/

27. Jarvis MJ, Tunstall-Pedoe H, Feyerabend C, et al. Comparison of tests used to distinguish smokers from nonsmokers. Am J Public Health. 1987 Nov;77(11):1435–8.

28. Benowitz NL. Biomarkers of environmental tobacco smoke exposure. Environ Health Perspect. 1999 May;107(Suppl 2):349–55.

29. Honarmand M, Nakhaee A, Moradi M. Comparison of Salivary Cotinine Concentrations in Male Smokers and Smokeless Tobacco Users. Asian Pac J Cancer Prev APJCP. 2018;19(5):1363–6.

30. What to Know if You Have to Take a Nicotine Test [Internet].[cited 2020 Jul 20] WebMD. Available from: https://www.webmd.com/smoking-cessation/taking-nicotine-test#1

31. Sample Size Tables for Clinical Studies, 3rd Edition | Wiley [Internet]. Wiley.com. [cited 2020 Oct 7]. Available from: https://www.wiley.com/enus/Sample+Size+Tables+for+Clinical+Studies%2C+3rd+Edition-p-9781444357967

32. Mahapatra S, Panda R. Effectiveness of face-to-face counseling with follow-up in primary care settings in India. Tobacco Induced Diseases. 2018 Mar 1;16(1).

33. Bailey SR, Stevens VJ, Fortmann SP, et al. Long-Term Outcomes from Repeated Smoking Cessation Assistance in Routine Primary Care. Am J Health Promot AJHP. 2018 Sep;32(7):1582–90.

34. BendtsenM (2020) Heterogeneous treatment effects of a text messaging smoking cessation intervention among university students. PLoS ONE 15(3): e0229637. https://doi.org/ 10.1371/journal.pone.0229637

35. Mishra GA, Pimple SA, Shastri SS. An overview of the tobacco problem in India. Indian J Med Paediatr Oncol Off J Indian Soc Med Paediatr Oncol. 2012;33(3):139–45

36. Kaur J, Rinkoo AV. Public Health Perspectives of Smokeless Tobacco and Areca Nut Use in the COVID-19 Era. Nicotine & Tobacco Research. 2020 May 8;

37. WHO. Q&A: Tobacco and COVID-19 [Internet]. www.who.int. 2020 [cited 2020 Jul 20]. Available from: https://www.who.int/news-room/q-a-detail/q-a-on-tobacco-and-covid-19

For peer review only - http://bmjopen.bmj.com/site/about/guidelines.xhtml

	STUDY PERIOD					
	**Pre Enrolment**	**Enrolment**	**Allocation**	**Post Allocation**		**Close-out**
**Time point**	0	0	0	0	3 months	After 3 months
**ENROLMENT:**						
Eligibility screening		X				
Informed consent		X				
Randomization to treatment allocation			X			
**INTERVENTIONS:**						
Routine care	X					
Ten-minute face-to-face counselling				X		
Mobile message based counselling					X	
**ASSESSMENTS:**						
Demographic		X				
Baseline assessment		X				
Mid-line assessment – Qualitative assessment at 1.5 months of recruitment					X	
End-line assessment						X
Saliva Cotinine assessment						X
Qualitative assessment with drop outs						X
Qualitative assessment with participants who successfully completed the follow-up						X

Table 1: SPIRIT figure illustrating the phases of CERTAIN trial and data collection time points

Figure 1: Trial schema showing the pathway for patients' recruitment process in CERTAIN study

For peer review only

For peer review only - http://bmjopen.bmj.com/site/about/guidelines.xhtml

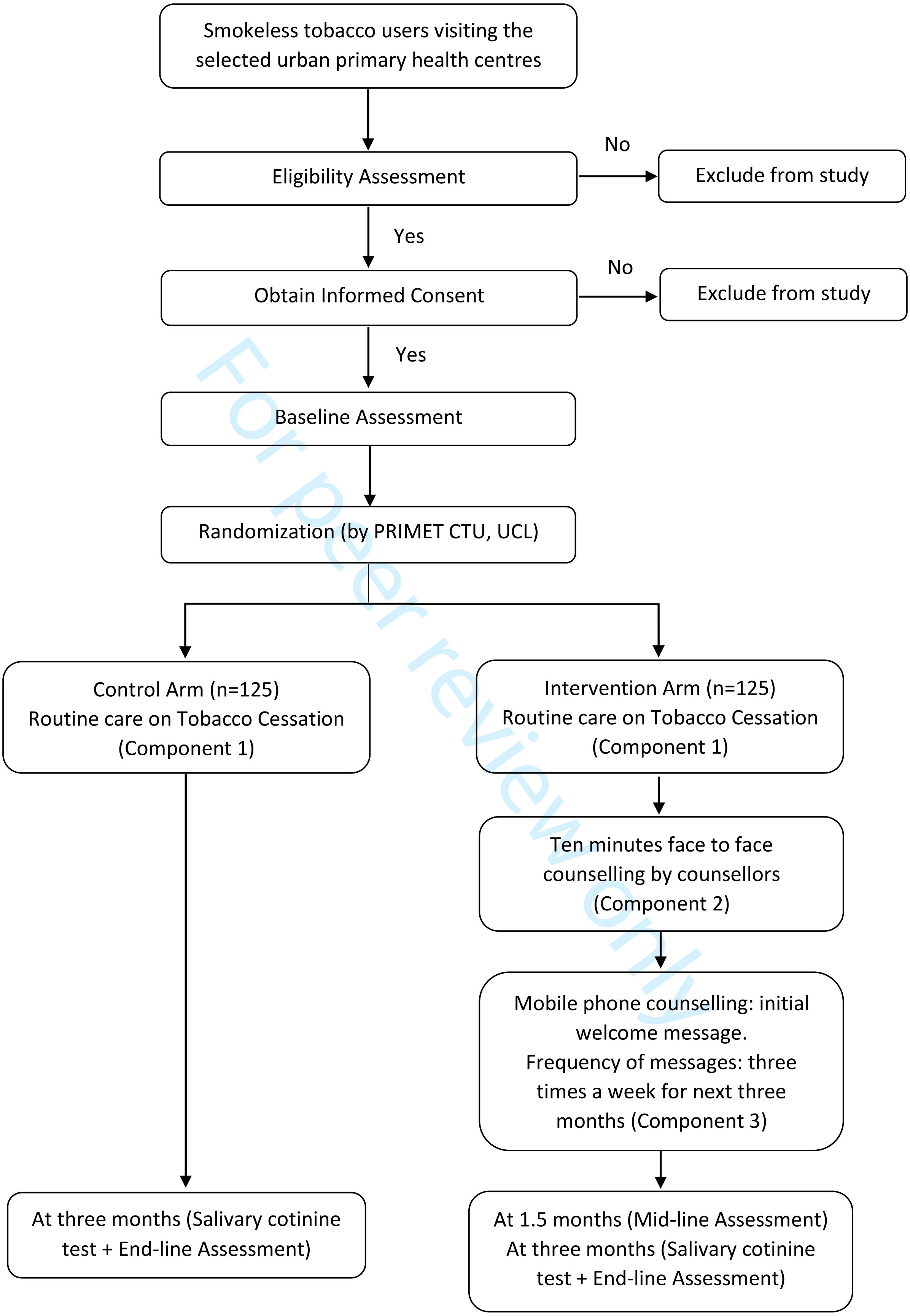

For peer review only

Appendix A: Details about the Formative Research phase of CERTAIN Study

Qualitative research methods will be used in the formative phase. In-Depth Interviews (IDIs) will be conducted with smokeless tobacco (SLT) users as well as primary care physicians present in UPHCs and Focus Group Discussions (FGDs) will be conducted with tobacco cessation counsellors. The formative research study will be based on a maximum variation sampling. A maximum variation sample will be constructed by identifying key dimensions of variations and then finding participants that vary from each other as much as possible. This sampling will yield detailed descriptions of each respondent, useful for documenting uniqueness, and this will allow researchers to identify common themes that are evident across the heterogeneous group of study participants. The variation will cover the socio-demographic characteristics of the study participants. The formative phase will assist in finalising two components of the intervention, contents for face to face counselling and a mobile phone intervention (regular messages). The formative research will be conducted in three sequential phases.

Phases in Formative Research:
In Phase 1, IDIs will be conducted with a purposive sample of male (n=15) and female (n=15) SLT users or dual users visiting UPHCs during the study period. IDIs will also be conducted with primary care physicians (n=5) present at these UPHCs. Participants' views on the detail of text messages will be explored. These views will inform the final text messages deemed suitable for tobacco cessation support. The messages will be tailored to users' health conditions and will use local terms, dialects and opinions of various stakeholders.

In Phase 2A, two FGDs will be conducted each with six primary care staff (ANM, ASHA, pharmacists, staff nurse, counsellors) who will be employed in the urban health clinic for more than a year and who show willingness to participate. Focus groups will explore the perspective of participants' regarding the preliminary content and structure of messages, use of tailored messages addressing the risk of tobacco for specific diseases, associated comorbidities, risks of tobacco usage in these comorbid conditions, readiness to quit stage and withdrawal symptoms.

In Phase 2B, developed text messages will undergo validation. The validation study will be conducted with SLT users visiting UPHCs. Each will be given a validation tool (developed specifically for this study) which will be used to score each of the newly developed messages. The messages that get the highest score will be adopted as a part of the intervention. IDIs will also be conducted with these tobacco users to gain their views and opinions regarding developed text messages.

Phase 3, will be conducted at two-time points (i.e., mid-line and end-line) in the trial follow-up period. At mid-line (1.5 months), IDIs will be conducted with SLT users to assess the appropriate engagement of the text messages. At end of three-months, IDIs will be conducted with SLT users who complete the follow-up period as well as with the SLT users who drop out of the study. Perceptions about appropriate engagement and satisfaction with text messages will be captured at this point.

The IDIs and FGDs will be conducted by trained qualitative research team members. Each team will have a moderator and note-taker. All interviews will be audio-recorded (with participants' consent) and transcribed. The transcriptions will be analysed using a qualitative framework analysis method. All transcripts will be reviewed and coded by two members of the research team to minimize subjective interpretations. We will use Atlas.ti (Version 8) software to evaluate the data.

Training of qualitative research team: All members of the qualitative research team participating in the study at each UPHCs will be trained. The training of qualitative researchers will include how to introduce the study to potential participants, obtaining verbal and written consent, conducting IDIs and FGDs, confidentiality and data transcription/translation/storing/handling.

For peer review only

SPIRIT

STANDARD PROTOCOL ITEMS: RECOMMENDATIONS FOR INTERVENTIONAL TRIALS

SPIRIT 2013 Checklist: Recommended items to address in a clinical trial protocol and related documents*

Section/item	Item No	Description	Yes/No	Page No
**Administrative information**				
Title	1	Descriptive title identifying the study design, population, interventions, and, if applicable, trial acronym	Yes	1
Trial registration	2a	Trial identifier and registry name. If not yet registered, name of intended registry	Yes	2
	2b	All items from the World Health Organization Trial Registration Data Set	Yes	2
Protocol version	3	Date and version identifier	Yes	1-11
Funding	4	Sources and types of financial, material, and other support	Yes	9
Roles and responsibilities	5a	Names, affiliations, and roles of protocol contributors	Yes	1
	5b	Name and contact information for the trial sponsor	Yes	9
	5c	Role of study sponsor and funders, if any, in study design; collection, management, analysis, and interpretation of data; writing of the report; and the decision to submit the report for publication, including whether they will have ultimate authority over any of these activities	N/A	N/A
	5d	Composition, roles, and responsibilities of the coordinating centre, steering committee, endpoint adjudication committee, data management team, and other individuals or groups overseeing the trial, if applicable (see Item 21a for data monitoring committee)	N/A	N/A

For peer review only

**Introduction**				
Background and rationale	6a	Description of research question and justification for undertaking the trial, including summary of relevant studies (published and unpublished) examining benefits and harms for each intervention	Yes	3-4
	6b	Explanation for choice of comparators	Yes	4
Objectives	7	Specific objectives or hypotheses	Yes	4
Trial design	8	Description of trial design including type of trial (eg, parallel group, crossover, factorial, single group), allocation ratio, and framework (eg, superiority, equivalence, noninferiority, exploratory)	Yes	4
**Methods: Participants, interventions, and outcomes**				
Study setting	9	Description of study settings (eg, community clinic, academic hospital) and list of countries where data will be collected. Reference to where list of study sites can be obtained	Yes	4
Eligibility criteria	10	Inclusion and exclusion criteria for participants. If applicable, eligibility criteria for study centres and individuals who will perform the interventions (eg, surgeons, psychotherapists)	Yes	4-5
Interventions	11a	Interventions for each group with sufficient detail to allow replication, including how and when they will be administered	Yes	5
	11b	Criteria for discontinuing or modifying allocated interventions for a given trial participant (eg, drug dose change in response to harms, participant request, or improving/worsening disease)	N/A	N/A
	11c	Strategies to improve adherence to intervention protocols, and any procedures for monitoring adherence (eg, drug tablet return, laboratory tests)	N/A	N/A

For peer review only

	11d	Relevant concomitant care and interventions that are permitted or prohibited during the trial	N/A	N/A
Outcomes	12	Primary, secondary, and other outcomes, including the specific measurement variable (eg, systolic blood pressure), analysis metric (eg, change from baseline, final value, time to event), method of aggregation (eg, median, proportion), and time point for each outcome. Explanation of the clinical relevance of chosen efficacy and harm outcomes is strongly recommended	Yes	5-6
Participant timeline	13	Time schedule of enrolment, interventions (including any run-ins and washouts), assessments, and visits for participants. A schematic diagram is highly recommended (see Figure)	Yes	12
Sample size	14	Estimated number of participants needed to achieve study objectives and how it was determined, including clinical and statistical assumptions supporting any sample size calculations	Yes	6
Recruitment	15	Strategies for achieving adequate participant enrolment to reach target sample size	Yes	6
**Methods: Assignment of interventions (for controlled trials)**				
Allocation:				
Sequence generation	16a	Method of generating the allocation sequence (eg, computer-generated random numbers), and list of any factors for stratification. To reduce predictability of a random sequence, details of any planned restriction (eg, blocking) should be provided in a separate document that is unavailable to those who enrol participants or assign interventions	Yes	6
Allocation concealment mechanism	16b	Mechanism of implementing the allocation sequence (eg, central telephone; sequentially numbered, opaque, sealed envelopes), describing any steps to conceal the sequence until interventions are assigned	Yes	6

Implementation	16c	Who will generate the allocation sequence, who will enrol participants, and who will assign participants to interventions	Yes	6
Blinding (masking)	17a	Who will be blinded after assignment to interventions (eg, trial participants, care providers, outcome assessors, data analysts), and how	Yes	6
	17b	If blinded, circumstances under which unblinding is permissible, and procedure for revealing a participant's allocated intervention during the trial	N/A	N/A
**Methods: Data collection, management, and analysis**				
Data collection methods	18a	Plans for assessment and collection of outcome, baseline, and other trial data, including any related processes to promote data quality (eg, duplicate measurements, training of assessors) and a description of study instruments (eg, questionnaires, laboratory tests) along with their reliability and validity, if known. Reference to where data collection forms can be found, if not in the protocol	Yes	6
	18b	Plans to promote participant retention and complete follow-up, including list of any outcome data to be collected for participants who discontinue or deviate from intervention protocols	Yes	6
Data management	19	Plans for data entry, coding, security, and storage, including any related processes to promote data quality (eg, double data entry; range checks for data values). Reference to where details of data management procedures can be found, if not in the protocol	Yes	7
Statistical methods	20a	Statistical methods for analysing primary and secondary outcomes. Reference to where other details of the statistical analysis plan can be found, if not in the protocol	Yes	7
	20b	Methods for any additional analyses (eg, subgroup and adjusted analyses)	Yes	7
	20c	Definition of analysis population relating to protocol non-adherence (eg, as randomised analysis), and any statistical methods to handle missing data (eg, multiple imputation)	Yes	7

**Methods: Monitoring**				
Data monitoring	21a	Composition of data monitoring committee (DMC); summary of its role and reporting structure; statement of whether it is independent from the sponsor and competing interests; and reference to where further details about its charter can be found, if not in the protocol. Alternatively, an explanation of why a DMC is not needed	N/A	N/A
	21b	Description of any interim analyses and stopping guidelines, including who will have access to these interim results and make the final decision to terminate the trial	N/A	N/A
Harms	22	Plans for collecting, assessing, reporting, and managing solicited and spontaneously reported adverse events and other unintended effects of trial interventions or trial conduct	Yes	9
Auditing	23	Frequency and procedures for auditing trial conduct, if any, and whether the process will be independent from investigators and the sponsor	N/A	N/A
**Ethics and dissemination**				
Research ethics approval	24	Plans for seeking research ethics committee/institutional review board (REC/IRB) approval	Yes	9
Protocol amendments	25	Plans for communicating important protocol modifications (eg, changes to eligibility criteria, outcomes, analyses) to relevant parties (eg, investigators, REC/IRBs, trial participants, trial registries, journals, regulators)	N/A	N/A
Consent or assent	26a	Who will obtain informed consent or assent from potential trial participants or authorised surrogates, and how (see Item 32)	Yes	9
	26b	Additional consent provisions for collection and use of participant data and biological specimens in ancillary studies, if applicable	N/A	N/A
Confidentiality	27	How personal information about potential and enrolled participants will be collected, shared, and maintained in order to protect confidentiality before, during, and after the trial	Yes	9

For peer review only - http://bmjopen.bmj.com/site/about/guidelines.xhtml

Declaration of interests	28	Financial and other competing interests for principal investigators for the overall trial and each study site	Yes	9
Access to data	29	Statement of who will have access to the final trial dataset, and disclosure of contractual agreements that limit such access for investigators	Yes	9
Ancillary and post-trial care	30	Provisions, if any, for ancillary and post-trial care, and for compensation to those who suffer harm from trial participation	N/A	N/A
Dissemination policy	31a	Plans for investigators and sponsor to communicate trial results to participants, healthcare professionals, the public, and other relevant groups (eg, via publication, reporting in results databases, or other data sharing arrangements), including any publication restrictions	Yes	4
	31b	Authorship eligibility guidelines and any intended use of professional writers	Yes	8
	31c	Plans, if any, for granting public access to the full protocol, participant-level dataset, and statistical code	N/A	N/A
**Appendices**				
Informed consent materials	32	Model consent form and other related documentation given to participants and authorised surrogates	Yes	Appendix B
Biological specimens	33	Plans for collection, laboratory evaluation, and storage of biological specimens for genetic or molecular analysis in the current trial and for future use in ancillary studies, if applicable	N/A	N/A

*It is strongly recommended that this checklist be read in conjunction with the SPIRIT 2013 Explanation & Elaboration for important clarification on the items. Amendments to the protocol should be tracked and dated. The SPIRIT checklist is copyrighted by the SPIRIT Group under the Creative Commons "Attribution-NonCommercial-NoDerivs 3.0 Unported" license.

For peer review only

BMJ Open

Exploratory randomized trial of face to face and mobile phone counselling against usual care for tobacco cessation in Indian primary care: a randomized controlled trial protocol for project CERTAIN.

Journal:	*BMJ Open*
Manuscript ID	bmjopen-2021-048628.R2
Article Type:	Protocol
Date Submitted by the Author:	23-Oct-2021
Complete List of Authors:	Panda, Rajmohan; Public Health Foundation of India, research<br>Omar, Rumana; University College London,<br>Hunter, Rachael; University College London, Research Dept of Primary Care and Population Health<br>Prabhu, Rajath R; Public Health Foundation of India, research<br>Mishra, Arti; Independent Consultant, Research<br>Nazareth, Irwin; UCL, Primary Care and Population Health
**Primary Subject Heading**:	Public health
Secondary Subject Heading:	Epidemiology, Smoking and tobacco
Keywords:	Protocols & guidelines < HEALTH SERVICES ADMINISTRATION & MANAGEMENT, PRIMARY CARE, QUALITATIVE RESEARCH, PUBLIC HEALTH

SCHOLARONE™
Manuscripts

Running Title: Counselling intErvention foR smokeless Tobacco cessAtion in INdian primary care: The CERTAIN study

Title: Exploratory randomized trial of face to face and mobile phone counselling against usual care for tobacco cessation in Indian primary care: a randomized controlled trial protocol for project CERTAIN.

Correspondence to: Dr Rajmohan Panda, raj.panda@phfi.org, Public Health Foundation of India, Plot Number 47, Sector 44, Gurugram, Haryana, India

Rajmohan Panda, Research Division, Public Health Foundation of India, Plot Number 47, Sector 44, Gurugram, Haryana, India

Rumana Omar, Department of Statistical Science, University College London, Gower Street, London, WC1E 6BT, United Kingdom

Rachael Hunter, Department of Primary Care and Population Sciences, University College London, Gower Street, London, WC1E 6BT, United Kingdom

Rajath R Prabhu, Research Division, Public Health Foundation of India, Plot Number 47, Sector 44, Gurugram, Haryana, India

Arti Mishra, Research, Independent Consultant

Irwin Nazareth, Department of Primary Care and Population Sciences, University College London, Gower Street, London, WC1E 6BT, United Kingdom

Word Count: 3491 (excluding title page, abstract, references, figures and tables)

Keywords/Phrases: tobacco cessation; smokeless tobacco; mobile counselling; primary care; formative research; exploratory randomized controlled trial

For peer review only

For peer review only - http://bmjopen.bmj.com/site/about/guidelines.xhtml

Abstract

Introduction: Despite widespread use of smokeless tobacco products by people in within the Indian sub-continent, there is little awareness amongst Indians of its health hazards when compared to smoked tobacco. We hypothesize that mobile phone counselling will be feasible, and effective for smokeless tobacco cessation intervention in India. This paper presents the protocol of the development and conduct of an exploratory trial before progression to a full randomized controlled trial.

Methods and Analysis: An exploratory randomized controlled trial will be conducted in urban primary health centres in the state of Odisha, India. A total of 250 smokeless tobacco users will be recruited to the study (125 in each arm). Participants in the intervention arm will receive routine care together with a face-to-face counselling intervention followed by advice and reminder mobile messages. The control arm will receive routine care, delivered by a primary care physician based on 'Ask' and 'Advice'. All participants will be followed up for three months from the first counselling session. The primary outcome of this trial is to assess the feasibility to carry out a full randomized controlled trial.

Ethics and Dissemination: Ethical approvals were obtained from the Institutional Ethics Committee of Public Health Foundation of India, Health Ministry's Screening Committee – ICMR, Odisha State Ethics Board, and also from University College London Research Ethics Committee, UK. The study findings will be published in a peer-reviewed scientific journal.

Trial Registration Number: CTRI Registration Number CTRI/2019/05/019484; dated May 31, 2019 (http://ctri.nic.in/Clinicaltrials/pmaindet2.php?trialid=30948&EncHid=&userName=Tobacco%20cessation)

Article Summary:

Strengths and limitations of this study:

- Evidence on the effectiveness of smokeless tobacco cessation intervention in primary care is scarce. The results of this study will inform on the feasibility of conducting an effectiveness trial in the future.
- Randomization with allocation concealment, blinded assessment of outcome measures, and registration of the protocol in the trial registry will minimize potential bias.
- Providing training to health professionals will lead to an improved understanding of the delivery of low-cost tobacco cessation interventions in primary care.
- The primary care physicians may get unmasked to the allocation of the participants during the course of the trial as the participants visiting the UPHCs may reveal the information to the physicians. There is a chance of biased results if physicians may deliver repeated face to face counselling services.
- The inclusion criteria include SLT users having a mobile phone, this may exclude a section of the SLT users who do not have a personal mobile phone like very poor or the elderly who do not use a mobile phone.

For peer review only - http://bmjopen.bmj.com/site/about/guidelines.xhtml

Introduction

Tobacco use is responsible for almost eight million deaths each year or a death every six seconds.[1,2] About 80% of 1.3 billion tobacco consumers across the globe live in Low-and Middle-Income Countries (LMICs).[1] In LMICs like India, the tobacco problem is complex as the country has a diverse population with a mixture of cultures, religions, and practices. The majority of the tobacco users in India use a variety of tobacco products – combustible, non-combustible, or both. As per the Global Adult Tobacco Survey 2 (GATS 2), 28.6% of the adult population in India consumes tobacco (10.7% of smoking and 21.4% of smokeless) making it the second-largest consumer in the world.[3]

Widespread use of smokeless tobacco (SLT) products occurs in countries such as in India and Bangladesh accounting for 232 of 248 million SLT users worldwide (i.e. from 21 countries).[4] SLT products such as "*tambaaku*", "*gurkha*", "*zarda*", "*khaini*", "*mawa*" and "*pan masala*" are widely used in many states in India as SLT consumption is culturally accepted and is considered common practice.[5] In India, SLT use is double that of combustible tobacco, making it the single most common form of tobacco used by men and women (29.6% and 12.8% respectively).[5] According to GATS 2 survey, the state of Odisha was one where the largest proportion of adults consumed SLT products (43%) compared with the national average (21.4%).[6] Despite strong evidence of SLT[7] on cancer of the oral cavity, pharynx, oesophagus, and also responsible for a large proportion of tobacco-related cancers in India, there is a misconception that SLT is relatively harmless .[8]

Primary care health professionals, the first contact for patients accessing help, play a key role in the health education of the hazards of tobacco. They are best placed to counsel against tobacco use and promote cessation. Studies on brief interventions by healthcare providers are effective in motivating tobacco users to quit tobacco.[9,10] A systematic review on SLT cessation intervention from both high income countries (HICs) and LMICs showed that behavioural interventions are effective in achieving cessation.[11] However, the overburdened primary care physicians in India have limited time for counselling[2,12,13] and it is also difficult to get users to attend follow-up visits as journeys to primary care clinics would incur a loss of income.[12]

In India, mobile phones are widely used in both rural and urban settings. India with 1173.7 million (98.2%) mobile phone users is the second-largest user base in the world.[14] Mobile phone subscribers in India increased from 1187 million in June 2019 to 1195.2 million by September 2019.[14] A meta-analysis of 104 studies including randomized trials and quasi-randomized trials on the effectiveness of telephone counselling for smoking tobacco cessation showed significant but modest effect sizes in HICs.[15] Effectiveness increases with treatment intensity.[16] A review of 26 trials on smoking cessation demonstrated that automated text messaging interventions were more effective than minimal smoking cessation support (RR 1.54, 95% CI 1.19 to 2.00; I2 = 71%; 13 studies) and text messaging added to other smoking cessation interventions was more effective than other individual smoking cessation interventions (RR 1.59, 95% CI 1.09 to 2.33; I2 = 0%, 4 studies).[17] Another meta-analysis of 13 trials reported smoking quit rates with text messaging intervention were 35% higher than quit rates for controls (OR = 1.35, 95% CI = 1.23, 1.49).[18] Other reviews of studies from HICs suggest that such interventions can increase the chance of quitting smoking tobacco from 39% to 80%.[19,20] Studies on SLT cessation that included randomized controlled trials conducted in HICs as well as some LMICs showed that behavioural cessation interventions led to quit rates between 9% to 51.5% at six months.[11,21] There are however little data on the efficacy and effectiveness of such intervention combined with mobile phone-based counselling in LMIC like India. Improved participation in tobacco cessation programmes through the use of mobile phone technology can have a substantial impact on a population at risk even with a small effect size.[22]

For peer review only - http://bmjopen.bmj.com/site/about/guidelines.xhtml

There is an urgent need to test the effectiveness of the use of mobile phone messages for SLT cessation in the Indian primary care setting.[23] We hypothesize that a face-to-face intervention offered by a tobacco counsellor together with low-cost mobile-based counselling has the potential to deliver a high-quality cessation intervention to SLT users. Prior to that we need to test the feasibility and acceptability of recruitment, delivery of the intervention and the follow-up of participants within a randomized controlled trial.

Aim and Objectives

Our overarching future aim is to evaluate within a randomized controlled trial, the clinical effectiveness (as measured by tobacco cessation) and cost-effectiveness of a complex intervention of face-to-face counselling coupled with mobile phone messaging delivered to SLT users visiting Indian primary care clinics in addition to routine care provided by the primary care professional against routine care alone. Prior to undertaking a full trial, we aim to develop and finalise the intervention and then test its acceptability and feasibility. Specific objectives of this study are to assess within an exploratory randomized controlled trial, the proportion of:

1. all primary care smokeless tobacco user attendees approached to take part in the trial that consent to randomization.
2. those who are randomized to the intervention, that comply with the intervention.
3. those who are randomized on whom the follow-up data can be collected.
4. missing data on all research measures administered at baseline and three months that will be used to assess the primary and secondary outcomes for conducting effectiveness randomized controlled trial in future.

Methodology

Patient and Public Involvement

The patients and public were involved in the formulation of the study hypothesis from previous research studies.[24,25] The research questions, design and outcome measures were derived from previous interaction with different stakeholders including patient and public. Tobacco users and other stakeholders (including primary care physicians, counsellors) will be involved in the development of two components of the intervention i.e., face-to-face counselling and mobile phone messages. These will be done by in-depth interviews and focus group discussion in the formative phase of the study *(Appendix A)*. The summary of the results of the study will be made available to patients and public at the end of the project period.

Study Design

The study will be conducted in two different phases. The first phase is a formative phase *(Appendix A)* and the second phase is an exploratory, parallel-group, randomized controlled trial, with pre-and post-test assessments. The duration of the study will be 18 months. The first phase will last for 8 months whereas the second phase will occur over 10 months (see table 1). The study flowchart is provided in figure 1.

Study Setting

The study will be conducted in urban primary health centres (UPHCs). All UPHCs in Berhampur city of Ganjam district of Odisha will be selected for this study.

Study Population

The study participants will include adult (above 18 years) SLT users who visit UPHCs in Berhampur city, Odisha. Participants must be current SLT users (i.e., users in the last three months), aged 18 years and

For peer review only - http://bmjopen.bmj.com/site/about/guidelines.xhtml

above, have a mobile phone with a valid contact number, and willing to consent to participate in the study.

Participants below the age of 18 years, who do not have the mental capacity to consent, with illness limiting their adherence and follow-up within the study, will be excluded.

Interventions

The intervention arm includes routine care (component 1) along with a single ten-minute face-to-face intervention based on 5A's approach (component 2), followed by mobile phone counselling (component 3). The control arm will be the provision of routine care alone (i.e. component 1).

CERTAIN Intervention: The intervention will be a combination of the following components -

1) Component one or delivery of "routine care" as delivered by a primary care physician over one to two minutes. This component will be delivered to both the intervention and control arms of the study. This is based on 'Ask' and 'Advice'. Under 'Ask', the physician will ask about consumption of SLT and under 'Advice', the physician will advise participants about the benefits of complete abstinence in a clear personalized manner.

2) Component two will include a single ten-minute face-to-face intervention delivered by a practice-based counsellor. This component will include brief standardized advice provided to participants based on 5A's approach[26] to tobacco cessation. Under this approach the counsellor will 'Ask' the participants if they use SLT; 'Advice' them on the importance of quitting (following the same script of component 1). Additionally, they will 'Assess' their willingness to 'quit now'; offer them 'Assistance' in the form of Nicotine Replacement Therapy (NRT) and/or provide them with a referral for behavioural support; and 'Arrange' a follow-up to check on progress. This will conclude with their enrolment to receive Component three – mobile phone intervention.

3) Component three will also follow 5A's approach to tobacco cessation. The intervention will include an initial message after 48 hours of recruitment, to remind them to quit tobacco followed by messages occurring three times a week for the next three months. Each follow-up message will offer behavioural messages to support quitting; information about health risks of tobacco use and benefits of quitting, and advice on withdrawal symptoms and coping strategies.

Training of tobacco cessation counsellors delivering the intervention: Counsellors at UPHCs are allied healthcare workers and will be trained by the members of the research team to deliver counselling intervention. They will not be involved in data collection or data analysis. Their training will be done using a standardized training manual (hardcopy of study manual will be provided to these counsellors) to maintain the fidelity of the intervention.

Outcomes

The main outcomes expected from this exploratory trial include:

1. The proportion of all the tobacco users approached who consent to randomization;
2. The proportion of those randomized who comply with the intervention; and
3. The proportion of those randomized on whom follow-up data are collected at three months;

We will also assess the feasibility of assessing outcomes of primary interest in the main trial as listed below:

For peer review only - http://bmjopen.bmj.com/site/about/guidelines.xhtml

- "Self-reported tobacco abstinence" of seven days confirmed by a salivary cotinine test.
- Self-reported motivation and intention to quit.
- Cost-effectiveness as cost per disability-adjusted life year (DALY) averted.
- Cost-effectiveness as cost per quality-adjusted life year (QALY) gained.

The outcomes will be assessed at baseline on recruitment to the study and the end of three months. At baseline, socio-demographic details such as age, gender, educational level, marital status, religion, community, the economic status will be recorded. Variables related to tobacco consumption will be recorded including, the number of SLT products per day (frequency), age of initiation, number of previous cessation attempts, and longest period of cessation. The data on quit attempts, the status of tobacco use, and challenges will be recorded during baseline and end-point. The biochemical validation of self-reported tobacco abstinence for seven days will be assessed using salivary cotinine at the end of three months. The rapid test kit works through visual interpretation of colour developed on the kit and is instant in processing.[27,28] Participants will be approached by the trial team members to provide a small sample of saliva which will be collected through passive drooling. Collection, transportation, and disposition of the biological samples and the kit will be managed at the study site by the site coordinator and research team. Cotinine is a preferred marker for tobacco use because cotinine stays in the body much longer than nicotine. The presence of cotinine in saliva or urine is a reliable indicator of tobacco use. Cotinine in vivo has a half-life of about twenty hours and lasts for about 3-4 days in the saliva and urine.[29,30]

Sample Size

Based on the primary outcomes in this exploratory trial of recruitment to the study and attrition, a total of 250 participants (125 participants per group) is required to estimate an anticipated proportion of 50% recruitment of participants with a 95% confidence interval (CI) of 44% to 57% and 20% attrition at follow-up with a 95% CI of 15% to 25%. The sample size was calculated based on estimating proportions with a specified level of precision at the 95% level as measured by the width of the confidence interval using the Sample Size Tables for Clinical Studies software.[31]

Randomization and Allocation

Randomization will be done at the level of individual eligible participants who have provided informed consent. Randomization will be stratified by study practice site (UPHCs) using random permuted blocks of varying block sizes from 4 to 10, and a 1:1 allocation to intervention or control arm. A randomization list will be prepared by an independent statistician (from PRIMENT CTU, UCL) not involved with this study. This list will be mailed to an independent staff member at the study site in India who will be responsible for the allocation of participants to the respective intervention arm. The independent staff member will assign a unique identification number to each of the consented participants and will maintain a list of those allocated to the respective study arm. The updated list will then be sent to the randomization coordinator who will be responsible for overseeing the recruitment and randomization process. The randomization coordinator will remove all the participant identifiers from the list and finally send the list to the statistician for the purpose of analysis.

The trial will ensure a clear separation between staff that collect outcome data and those that deliver the intervention. Staff collecting outcome data will be blinded to the group assignment. None of the intervention staff, primary care physicians, and behavioural counsellors will collect outcome data. All investigators, staff, and participants will be kept masked to outcome measurements and any preliminary results emerging from the trial.

For peer review only - http://bmjopen.bmj.com/site/about/guidelines.xhtml

Data Management

Baseline and end-point data will be collected from participants at UPHC using surveys in paper format. This will be entered electronically in the database developed in MS Access (Microsoft Office 2019). The quality of the data will be monitored by a data supervisor for completeness, validity, and integrity. In case of incomplete data or inconsistent responses, the data supervisor will verify these with the study participants and update the entries in the database. The database will incorporate a range of inconsistency checks to limit data entry errors. Time-stamped audit trail will also be built in the database to independently record the date and time of operator entries and actions that create, modify, or delete electronic records.

Data Analysis

Statistical Analysis: Participant characteristics will be summarised using mean and standard deviation [SD] or median and interquartile range for continuous variables, and number & percentages for the categorical variables. The proportion of participants recruited and lost to follow-up at the end-point will be estimated with 95% confidence intervals. As part of the secondary analyses, logistic regression models will be used to estimate the intervention effects with 95% confidence intervals for the pre-specified outcomes, tobacco abstinence and self-reported motivation and intention to quit after adjusting for the stratification factor (UPHC) and baseline values of the outcome where it is available. These analyses will be done on an intention to treat (participants as randomized with available outcome data) basis and multiple imputation will be used to handle missing outcome values if considered appropriate. The extent of missing data for each variable and the percentage of participants adhering to the intervention will be reported. Attrition levels by randomized group and the characteristics of participants who are lost to follow-up will also be reported. All analyses will be done using STATA software version 17 (StataCorp. 2019. *Stata Statistical Software: Release 17*. College Station, TX: StataCorp LLC) or updated versions. A detailed statistical analysis plan will be drawn up nearer the analysis stage, prior to the database lock.

Economic Evaluation: The aim will be to evaluate the feasibility of calculating the cost-effectiveness of routine care along with intervention compared to routine care alone from an Indian health care cost perspective over three months. This will include the level of data completeness of self-completed questionnaires of tobacco-related health care resource use, estimation of the intervention, and routine care costs. The feasibility of calculating cost per DALY averted, particularly in relation to potential cases of cancer avoided, as part of the main trial will also be evaluated in addition to data requirements for a decision model to evaluate the lifetime cost-effectiveness of the intervention calculated as the cost per QALY gained.

Trial Status

The two major COVID-19 pandemic waves have affected India (wave one in March 2020 and wave two in April 2021). This has delayed the recruitment of the trial since out-patient departments (OPDs) were not operational or operating with limited staff. Our current plan is to start recruitment by mid-July 2021. As per the original proposal, the follow-up was for a period of six months, our current plan for follow-up is now for three months.

Discussion

To the best of our knowledge, this is the first study evaluating a complex low-cost intervention of two components i.e., face-to-face counselling along with mobile phone messaging counselling delivered to SLT users in low resource Indian settings. The first component of the intervention is based on 5A's approach which is known to be effective.[32] However, such a brief intervention may have a limited

For peer review only - http://bmjopen.bmj.com/site/about/guidelines.xhtml

effect if the person is not ready to quit on account of other factors associated with difficulty in quitting tobacco.[33] However, follow-up quitting advice to tobacco users can increase quit attempts and quit rates[32] and this will be achieved through, the mobile phone messaging. Mobile based interventions are widely used in the developed world and research on smoking cessation suggests that they can increase tobacco quitting by 25-50%.[20] A study conducted in Sweden assessed the heterogeneous treatment effects of text messaging intervention among smokers and found that the effect was less pronounced among the participants with stronger nicotine dependence.[34] Tailoring of such interventions for specific individuals needs to be considered, as each individual will need messaging according to their beliefs and their readiness to quit. The mobile messages in our study will be developed with this context in mind. To date, there have been no evaluations conducted to assess the effectiveness of such complex low-cost intervention in low resource settings and primary care. The development and delivery of comprehensive, tailored primary care behavioural and mobile phone health technology interventions will enhance the understanding of low-cost long-term prevention intervention for tobacco cessation. Recent evidence has emerged on the association of tobacco use with novel coronavirus disease.[35,36] The use of SLT often involves hand-to-mouth contact that can promote the spread of COVID-19. Another behaviour that can spread infections associated with the use of SLT is the spitting of or projection of excess saliva produced during the chewing process.[37] Whilst, the struggle with coronavirus infection control continues, we have an opportunity to expand cessation intervention specifically designed for SLT use and promote good infection control practice.

Strengths and Limitations

This study has many strengths. The results of this study will inform the feasibility of conducting effectiveness trials in the future. Incorporating messaging technology in routine clinical practice will be time-efficient as the messages can be delivered anywhere, at fixed times, and directly to the participant with negligible direct contact whilst also ensuring privacy. The impact of this research on the training of health professionals will lead to an improved understanding of the delivery of low-cost tobacco cessation interventions in primary care. There are however some limitations to the study. As the study is being conducted in urban health centres in Berhampur city, Odisha, the findings would not be generalisable to other healthcare settings as well as to the whole population of the Odisha state. The primary care physicians may get unmasked to the allocation of the participants during the course of the trial as the participants visiting the UPHCs may reveal the information to the physicians. There is a chance of biased results if physicians may deliver repeated face to face counselling services. The inclusion criteria include SLT users having a mobile phone, this may exclude a section of the SLT users who do not have a personal mobile phone like people of low socio-economic status or elderly who do not use a mobile phone. The follow up is only for three months. Longer follow-up assessments might provide different findings.

Research Impact

In this study, SLT cessation intervention will be developed using an innovative method including a formative research phase, where the SLT cessation intervention will be developed; and the exploratory randomized controlled trial phase, where the evaluation of this intervention will be conducted. The evidence from this exploratory study will inform the acceptability and feasibility of SLT cessation interventions in users of SLT and will inform the conduct of a larger multi-centric trial across several centres and countries. Findings from this study will provide insights for designing similar studies and appropriate interventions in tobacco cessation and non-communicable diseases. Since research in this area is in its infancy in LMIC, this work will provide an impetus for researchers working on tobacco cessation to generate new evidence and allow them to adapt the tools developed and piloted in this study in real time practice.

For peer review only - http://bmjopen.bmj.com/site/about/guidelines.xhtml

Contributions: All authors contributed to the manuscript. RP (PI of the study – India) and IN (PI of the study – UK) designed the study, led on writing, review and finalising the paper. RO provided statistical guidance. RH provided inputs on designing the economic evaluation section of the study. AM provided inputs on designing the formative research section of the study. RRP reviewed and assisted in writing the early drafts of the paper. All authors provided critical feedback and helped in shaping the manuscript.

Funding statement: This research work is funded by the Joint Global Health Trials Initiative MRC/DfID/Wellcome Trust (ref: MR/P021166/1)

Sponsor: This research work is sponsored by University College London, Gower Street, London, WC1E 6BT, United Kingdom

Competing interests: None declared

Ethics and Dissemination: Ethics approval has been obtained from the Institutional Ethical Committee at Public Health Foundation of India (PHFI) (ref: TRC-IEC-391/19; dated May 29, 2019). At the national level, ethical clearance has also been obtained from the Health Ministry's Screening Committee (HMSC), led by the Indian Council of Medical Research (ICMR) (ref: 2019-3581; dated December 11, 2019). Also, local level approval was obtained from the Odisha State Ethics Board (ref: 191/PMU/187/17; dated November 14, 2019). In the UK, ethical clearance was obtained by the UCL Research Ethics Committee (ref: 5686/001, dated October 1, 2019). The study has been registered at Clinical Trials Registry India (reference number CTRI/2019/05/019484). All the participants (participating in both the phases in this study) will be provided with a participant information sheet (PIS), providing details of the study. Following this, voluntary written consent for participation will also be taken from them *(Appendix B)*. All the data that will be collected for the study will be stripped of any personal identifiers and the data will be stored in PHFI's data repository. The data will only be accessible to the principal investigator and trial team analysing the data. To ensure confidentiality, data shared to project team members will be blinded for any identifying participant information. The findings of the study will be published in peer-reviewed journals.

Data sharing statement: The authors will make available data underpinning research publications at the time of such publication, or at any time prior to publication when reasonably requested, as well as any original software that may be required to view such data.

References

1. World Health Organization. Tobacco [Internet]. [cited 2020 Jul 20]Who.int. World Health Organization: WHO; 2018. Available from: https://www.who.int/news-room/fact-sheets/detail/tobacco

2. Amit S, Bhambal A, Saxena V et al. Tobacco cessation and counseling: a dentists' perspective in Bhopal city, Madhya Pradesh. Indian J Dent Res Off Publ Indian Soc Dent Res. 2011 Jun;22(3):400–3.

3. GATS 2 Global Adult Tobacco Survey [Internet]. [cited 2020 Jul 20] Available from: https://www.who.int/tobacco/surveillance/survey/gats/GATS_India_2016-17_FactSheet.pdf

4. GATS Atlas Online [Internet]. gatsatlas.org. [cited 2020 Jul 20]. Available from: http://gatsatlas.org/pdf/mobile/index.html#p=38

For peer review only

For peer review only - http://bmjopen.bmj.com/site/about/guidelines.xhtml

5. Murthy P, Subodh BN, Sinha D, et al. Smokeless tobacco (SLT) use and cessation in India: Lessons from user and health care provider perspectives. Asian J Psychiatry. 2018 Feb;32:137–42.

6. GATS 2 Global Adult Tobacco Survey [Internet]. [cited 2020 Jul 20]. Available from: https://tiss.edu/uploads/files/Odisha.pdf

7. Monika S, Dineshkumar T, Priyadharini S, et al. Smokeless Tobacco Products (STPs) Harbour Bacterial Populations with Potential for Oral Carcinogenicity. Asian Pac J Cancer Prev. 2020 Mar 1;21(3):815–24.

8. Raute LJ, Sansone G, Pednekar MS, et al. Knowledge of health effects and intentions to quit among smokeless tobacco users in India: findings from the International Tobacco Control Policy Evaluation (ITC) India Pilot Survey. Asian Pac J Cancer Prev APJCP. 2011;12(5):1233–8.

9. Gordon JS, Andrews JA, Crews KM, et al. The 5A's vs 3A's plus proactive quitline referral in private practice dental offices: preliminary results. Tob Control. 2007 Aug;16(4):285–8.

10. Gordon JS, Andrews JA, Albert DA, et al. Tobacco Cessation via Public Dental Clinics: Results of a Randomized Trial. Am J Public Health. 2010 Jul;100(7):1307–12.

11. Nethan ST, Sinha DN, Chandan K, et al. Smokeless tobacco cessation interventions: A systematic review. Indian J Med Res. 2018 Apr 1;148(4):396.

12. Champassak SL, Goggin K, Finocchario-Kessler S, et al. A qualitative assessment of provider perspectives on smoking cessation counselling. Journal of Evaluation in Clinical Practice. 2014 Jun 1;20(3):281–287.

13. Jradi H. Awareness, practices, and barriers regarding smoking cessation treatment among physicians in Saudi Arabia. J Addict Dis. 2017 Jan 2;36(1):53–9.

14. Bhawan M. The Indian Telecom Services Performance Indicators, July – September, 2019 [Internet]. [cited 2020 Jul 20] 2020 Jan. Available from: https://trai.gov.in/sites/default/files/PIR_08012020_0.pdf

15. Matkin W, Ordóñez-Mena JM, Hartmann-Boyce J. Telephone counselling for smoking cessation. Cochrane Database Syst Rev. 2019 May 02 [Internet].[cited 2020 May 24];(5). Available from: https://www.cochranelibrary.com/cdsr/doi/10.1002/14651858.CD002850.pub4/full

16. Fiore M, United States. Tobacco Use And Dependence Guideline Panel. Treating tobacco use and dependence : 2008 update. Darby, Pa.: Diane Pub. Co; 2009.

17. Whittaker R, McRobbie H, Bullen C, et al. Mobile phone text messaging and app-based interventions for smoking cessation. Cochrane Database Syst Rev. 2019 Oct 22;10:CD006611

18. Spohr SA, Nandy R, Gandhiraj D, et al. Efficacy of SMS Text Message Interventions for Smoking Cessation: A Meta-Analysis. J Subst Abuse Treat. 2015 Sep;56:1–10.

19. Whitehouse E, Lai J, Golub JE, et al. A systematic review of the effectiveness of smoking cessation interventions among patients with tuberculosis. Public Health Action. 2018 Jun 21;8(2):37–49.

20. Stead LF, Hartmann-Boyce J, Perera R, et al. Telephone counselling for smoking cessation. Cochrane Database Syst Rev. 2013 Aug 12;(8):CD002850.

21. Messina J, Freeman C, Rees A, et al. Smokeless tobacco: South Asians -Reviews 1 and 2 - Effectiveness and contextual factors Systematic review of effectiveness of smokeless tobacco

For peer review only - http://bmjopen.bmj.com/site/about/guidelines.xhtml

interventions for South Asians and a review of contextual factors relating to smokeless tobacco among South Asian users and health care providers [Internet]. [cited 2020 Jul 20]. Available from: https://www.nice.org.uk/guidance/ph39/documents/smokeless-tobacco-cessation-south-asians-evidence-reviews-1-and-22

22. Baskerville NB, Struik LL, Hammond D, et al. Effect of a mobile phone intervention on quitting smoking in a young adult population of smokers: randomized controlled trial study protocol. JMIR Res Protoc. 2015 Jan 19;4(1):e10.

23. IndianJMedRes1406707-3538149_094941.pdf [Internet]. [cited 2020 Jul 20]. Available from: http://www.ijmr.org.in/temp/IndianJMedRes1406707-3538149_094941.pdf

24. Panda R, Persai D, Venkatesan S. Missed opportunities for brief intervention in tobacco control in primary care: patients' perspectives from primary health care settings in India. BMC Health Services Research. 2015 Feb 1;15(1).

25. Panda R, Persai D, Venkatesan S, Ahluwalia JS. Physician and patient concordance of report of tobacco cessation intervention in primary care in India. BMC Public Health. 2015 May 2;15(1).

26. Tobacco Use and Dependence Guideline Panel. Treating Tobacco Use and Dependence: 2008 Update [Internet]. Nih.gov. US Department of Health and Human Services; 2008. Available from: https://www.ncbi.nlm.nih.gov/books/NBK63952/

27. Jarvis MJ, Tunstall-Pedoe H, Feyerabend C, et al. Comparison of tests used to distinguish smokers from nonsmokers. Am J Public Health. 1987 Nov;77(11):1435–8.

28. Benowitz NL. Biomarkers of environmental tobacco smoke exposure. Environ Health Perspect. 1999 May;107(Suppl 2):349–55.

29. Honarmand M, Nakhaee A, Moradi M. Comparison of Salivary Cotinine Concentrations in Male Smokers and Smokeless Tobacco Users. Asian Pac J Cancer Prev APJCP. 2018;19(5):1363–6.

30. What to Know if You Have to Take a Nicotine Test [Internet].[cited 2020 Jul 20] WebMD. Available from: https://www.webmd.com/smoking-cessation/taking-nicotine-test#1

31. Sample Size Tables for Clinical Studies, 3rd Edition | Wiley [Internet]. Wiley.com. [cited 2020 Oct 7]. Available from: https://www.wiley.com/enus/Sample+Size+Tables+for+Clinical+Studies%2C+3rd+Edition-p-9781444357967

32. Mahapatra S, Panda R. Effectiveness of face-to-face counseling with follow-up in primary care settings in India. Tobacco Induced Diseases. 2018 Mar 1;16(1).

33. Bailey SR, Stevens VJ, Fortmann SP, et al. Long-Term Outcomes from Repeated Smoking Cessation Assistance in Routine Primary Care. Am J Health Promot AJHP. 2018 Sep;32(7):1582–90.

34. BendtsenM (2020) Heterogeneous treatment effects of a text messaging smoking cessation intervention among university students. PLoS ONE 15(3): e0229637. https://doi.org/10.1371/journal.pone.0229637

35. Mishra GA, Pimple SA, Shastri SS. An overview of the tobacco problem in India. Indian J Med Paediatr Oncol Off J Indian Soc Med Paediatr Oncol. 2012;33(3):139–45

For peer review only

For peer review only - http://bmjopen.bmj.com/site/about/guidelines.xhtml

36. Kaur J, Rinkoo AV. Public Health Perspectives of Smokeless Tobacco and Areca Nut Use in the COVID-19 Era. Nicotine & Tobacco Research. 2020 May 8;

37. WHO. Q&A: Tobacco and COVID-19 [Internet]. www.who.int. 2020 [cited 2020 Jul 20]. Available from: https://www.who.int/news-room/q-a-detail/q-a-on-tobacco-and-covid-19

	STUDY PERIOD					
	**Pre Enrolment**	**Enrolment**	**Allocation**	**Post Allocation**		**Close-out**
**Time point**	0	0	0	0	3 months	After 3 months
**ENROLMENT:**						
Eligibility screening		X				
Informed consent		X				
Randomization to treatment allocation			X			
**INTERVENTIONS:**						
Routine care	X					
Ten-minute face-to-face counselling				X		
Mobile message based counselling					X	
**ASSESSMENTS:**						
Demographic		X				
Baseline assessment		X				
Mid-line assessment – Qualitative assessment at 1.5 months of recruitment					X	
End-line assessment						X
Saliva Cotinine assessment						X
Qualitative assessment with drop outs						X
Qualitative assessment with participants who successfully completed the follow-up						X

Table 1: SPIRIT figure illustrating the phases of CERTAIN trial and data collection time points

Figure 1: Trial schema showing the pathway for patients' recruitment process in CERTAIN study

For peer review only - http://bmjopen.bmj.com/site/about/guidelines.xhtml

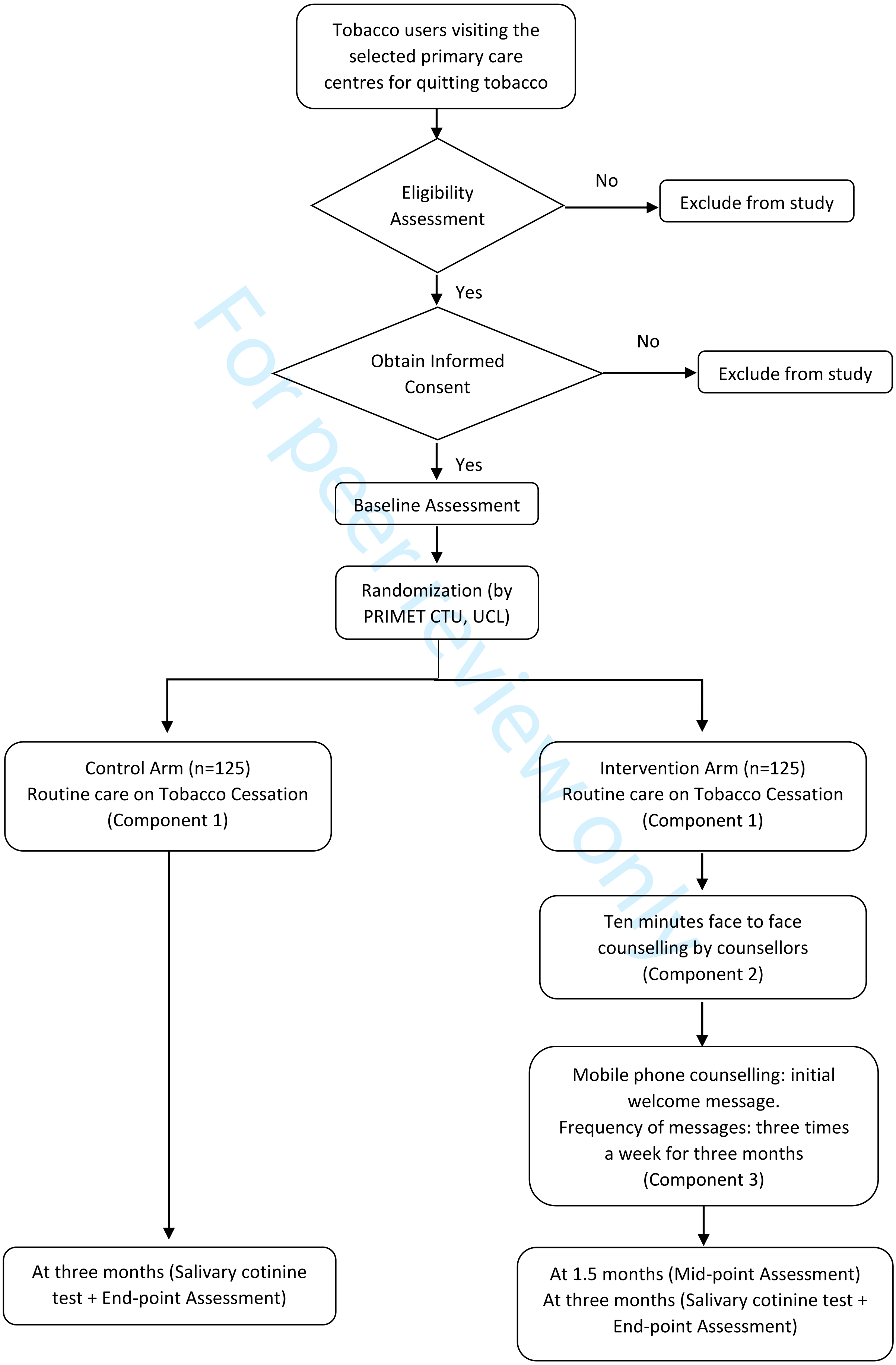

For peer review only

Appendix A: Details about the Formative Research phase of CERTAIN Study

Qualitative research methods will be used in the formative phase. In-Depth Interviews (IDIs) will be conducted with smokeless tobacco (SLT) users as well as primary care physicians present in UPHCs and Focus Group Discussions (FGDs) will be conducted with tobacco cessation counsellors. The formative research study will be based on a maximum variation sampling. A maximum variation sample will be constructed by identifying key dimensions of variations and then finding participants that vary from each other as much as possible. This sampling will yield detailed descriptions of each respondent, useful for documenting uniqueness, and this will allow researchers to identify common themes that are evident across the heterogeneous group of study participants. The variation will cover the socio-demographic characteristics of the study participants. The formative phase will assist in finalising two components of the intervention, contents for face to face counselling and a mobile phone intervention (regular messages). The formative research will be conducted in three sequential phases.

Phases in Formative Research:

In Phase 1, IDIs will be conducted with a purposive sample of male (n=15) and female (n=15) SLT users or dual users visiting UPHCs during the study period. IDIs will also be conducted with primary care physicians (n=5) present at these UPHCs. Participants' views on the detail of text messages will be explored. These views will inform the final text messages deemed suitable for tobacco cessation support. The messages will be tailored to users' health conditions and will use local terms, dialects and opinions of various stakeholders.

In Phase 2A, two FGDs will be conducted each with six primary care staff (ANM, ASHA, pharmacists, staff nurse, counsellors) who will be employed in the urban health clinic for more than a year and who show willingness to participate. Focus groups will explore the perspective of participants' regarding the preliminary content and structure of messages, use of tailored messages addressing the risk of tobacco for specific diseases, associated comorbidities, risks of tobacco usage in these comorbid conditions, readiness to quit stage and withdrawal symptoms.

In Phase 2B, developed text messages will undergo validation. The validation study will be conducted with SLT users visiting UPHCs. Each will be given a validation tool (developed specifically for this study) which will be used to score each of the newly developed messages. The messages that get the highest score will be adopted as a part of the intervention. IDIs will also be conducted with these tobacco users to gain their views and opinions regarding developed text messages.

Phase 3, will be conducted at two-time points (i.e., mid-line and end-line) in the trial follow-up period. At mid-line (1.5 months), IDIs will be conducted with SLT users to assess the appropriate engagement of the text messages. At end of three-months, IDIs will be conducted with SLT users who complete the follow-up period as well as with the SLT users who drop out of the study. Perceptions about appropriate engagement and satisfaction with text messages will be captured at this point.

The IDIs and FGDs will be conducted by trained qualitative research team members. Each team will have a moderator and note-taker. All interviews will be audio-recorded (with participants' consent) and transcribed. The transcriptions will be analysed using a qualitative framework analysis method. All transcripts will be reviewed and coded by two members of the research team to minimize subjective interpretations. We will use Atlas.ti (Version 8) software to evaluate the data.

Training of qualitative research team: All members of the qualitative research team participating in the study at each UPHCs will be trained. The training of qualitative researchers will include how to introduce the study to potential participants, obtaining verbal and written consent, conducting IDIs and FGDs, confidentiality and data transcription/translation/storing/handling.

For peer review only

STANDARD PROTOCOL ITEMS: RECOMMENDATIONS FOR INTERVENTIONAL TRIALS

SPIRIT 2013 Checklist: Recommended items to address in a clinical trial protocol and related documents*

Section/item	Item No	Description	Yes/No	Page No
**Administrative information**				
Title	1	Descriptive title identifying the study design, population, interventions, and, if applicable, trial acronym	Yes	1
Trial registration	2a	Trial identifier and registry name. If not yet registered, name of intended registry	Yes	2
	2b	All items from the World Health Organization Trial Registration Data Set	Yes	2
Protocol version	3	Date and version identifier	Yes	1-12
Funding	4	Sources and types of financial, material, and other support	Yes	9
Roles and responsibilities	5a	Names, affiliations, and roles of protocol contributors	Yes	1
	5b	Name and contact information for the trial sponsor	Yes	9
	5c	Role of study sponsor and funders, if any, in study design; collection, management, analysis, and interpretation of data; writing of the report; and the decision to submit the report for publication, including whether they will have ultimate authority over any of these activities	N/A	N/A
	5d	Composition, roles, and responsibilities of the coordinating centre, steering committee, endpoint adjudication committee, data management team, and other individuals or groups overseeing the trial, if applicable (see Item 21a for data monitoring committee)	N/A	N/A

For peer review only

**Introduction**				
Background and rationale	6a	Description of research question and justification for undertaking the trial, including summary of relevant studies (published and unpublished) examining benefits and harms for each intervention	Yes	3-4
	6b	Explanation for choice of comparators	Yes	4
Objectives	7	Specific objectives or hypotheses	Yes	4
Trial design	8	Description of trial design including type of trial (eg, parallel group, crossover, factorial, single group), allocation ratio, and framework (eg, superiority, equivalence, noninferiority, exploratory)	Yes	4
**Methods: Participants, interventions, and outcomes**				
Study setting	9	Description of study settings (eg, community clinic, academic hospital) and list of countries where data will be collected. Reference to where list of study sites can be obtained	Yes	4
Eligibility criteria	10	Inclusion and exclusion criteria for participants. If applicable, eligibility criteria for study centres and individuals who will perform the interventions (eg, surgeons, psychotherapists)	Yes	4-5
Interventions	11a	Interventions for each group with sufficient detail to allow replication, including how and when they will be administered	Yes	5
	11b	Criteria for discontinuing or modifying allocated interventions for a given trial participant (eg, drug dose change in response to harms, participant request, or improving/worsening disease)	N/A	N/A
	11c	Strategies to improve adherence to intervention protocols, and any procedures for monitoring adherence (eg, drug tablet return, laboratory tests)	N/A	N/A

For peer review only - http://bmjopen.bmj.com/site/about/guidelines.xhtml

	11d	Relevant concomitant care and interventions that are permitted or prohibited during the trial	N/A	N/A
Outcomes	12	Primary, secondary, and other outcomes, including the specific measurement variable (eg, systolic blood pressure), analysis metric (eg, change from baseline, final value, time to event), method of aggregation (eg, median, proportion), and time point for each outcome. Explanation of the clinical relevance of chosen efficacy and harm outcomes is strongly recommended	Yes	5-6
Participant timeline	13	Time schedule of enrolment, interventions (including any run-ins and washouts), assessments, and visits for participants. A schematic diagram is highly recommended (see Figure)	Yes	Figure 1 (Image)
Sample size	14	Estimated number of participants needed to achieve study objectives and how it was determined, including clinical and statistical assumptions supporting any sample size calculations	Yes	6
Recruitment	15	Strategies for achieving adequate participant enrolment to reach target sample size	Yes	6
**Methods: Assignment of interventions (for controlled trials)**				
Allocation:				
Sequence generation	16a	Method of generating the allocation sequence (eg, computer-generated random numbers), and list of any factors for stratification. To reduce predictability of a random sequence, details of any planned restriction (eg, blocking) should be provided in a separate document that is unavailable to those who enrol participants or assign interventions	Yes	6
Allocation concealment mechanism	16b	Mechanism of implementing the allocation sequence (eg, central telephone; sequentially numbered, opaque, sealed envelopes), describing any steps to conceal the sequence until interventions are assigned	Yes	6

Implementation	16c	Who will generate the allocation sequence, who will enrol participants, and who will assign participants to interventions	Yes	6
Blinding (masking)	17a	Who will be blinded after assignment to interventions (eg, trial participants, care providers, outcome assessors, data analysts), and how	Yes	6
	17b	If blinded, circumstances under which unblinding is permissible, and procedure for revealing a participant's allocated intervention during the trial	N/A	N/A
**Methods: Data collection, management, and analysis**				
Data collection methods	18a	Plans for assessment and collection of outcome, baseline, and other trial data, including any related processes to promote data quality (eg, duplicate measurements, training of assessors) and a description of study instruments (eg, questionnaires, laboratory tests) along with their reliability and validity, if known. Reference to where data collection forms can be found, if not in the protocol	Yes	6
	18b	Plans to promote participant retention and complete follow-up, including list of any outcome data to be collected for participants who discontinue or deviate from intervention protocols	Yes	6
Data management	19	Plans for data entry, coding, security, and storage, including any related processes to promote data quality (eg, double data entry; range checks for data values). Reference to where details of data management procedures can be found, if not in the protocol	Yes	7
Statistical methods	20a	Statistical methods for analysing primary and secondary outcomes. Reference to where other details of the statistical analysis plan can be found, if not in the protocol	Yes	7
	20b	Methods for any additional analyses (eg, subgroup and adjusted analyses)	Yes	7
	20c	Definition of analysis population relating to protocol non-adherence (eg, as randomised analysis), and any statistical methods to handle missing data (eg, multiple imputation)	Yes	7

**Methods: Monitoring**				
Data monitoring	21a	Composition of data monitoring committee (DMC); summary of its role and reporting structure; statement of whether it is independent from the sponsor and competing interests; and reference to where further details about its charter can be found, if not in the protocol. Alternatively, an explanation of why a DMC is not needed	N/A	N/A
	21b	Description of any interim analyses and stopping guidelines, including who will have access to these interim results and make the final decision to terminate the trial	N/A	N/A
Harms	22	Plans for collecting, assessing, reporting, and managing solicited and spontaneously reported adverse events and other unintended effects of trial interventions or trial conduct	Yes	9
Auditing	23	Frequency and procedures for auditing trial conduct, if any, and whether the process will be independent from investigators and the sponsor	N/A	N/A
**Ethics and dissemination**				
Research ethics approval	24	Plans for seeking research ethics committee/institutional review board (REC/IRB) approval	Yes	9
Protocol amendments	25	Plans for communicating important protocol modifications (eg, changes to eligibility criteria, outcomes, analyses) to relevant parties (eg, investigators, REC/IRBs, trial participants, trial registries, journals, regulators)	N/A	N/A
Consent or assent	26a	Who will obtain informed consent or assent from potential trial participants or authorised surrogates, and how (see Item 32)	Yes	9
	26b	Additional consent provisions for collection and use of participant data and biological specimens in ancillary studies, if applicable	N/A	N/A
Confidentiality	27	How personal information about potential and enrolled participants will be collected, shared, and maintained in order to protect confidentiality before, during, and after the trial	Yes	9

review only

Declaration of interests	28	Financial and other competing interests for principal investigators for the overall trial and each study site	Yes	9
Access to data	29	Statement of who will have access to the final trial dataset, and disclosure of contractual agreements that limit such access for investigators	Yes	9
Ancillary and post-trial care	30	Provisions, if any, for ancillary and post-trial care, and for compensation to those who suffer harm from trial participation	N/A	N/A
Dissemination policy	31a	Plans for investigators and sponsor to communicate trial results to participants, healthcare professionals, the public, and other relevant groups (eg, via publication, reporting in results databases, or other data sharing arrangements), including any publication restrictions	Yes	4
	31b	Authorship eligibility guidelines and any intended use of professional writers	Yes	8
	31c	Plans, if any, for granting public access to the full protocol, participant-level dataset, and statistical code	N/A	N/A
**Appendices**				
Informed consent materials	32	Model consent form and other related documentation given to participants and authorised surrogates	Yes	Appendix B
Biological specimens	33	Plans for collection, laboratory evaluation, and storage of biological specimens for genetic or molecular analysis in the current trial and for future use in ancillary studies, if applicable	N/A	N/A

*It is strongly recommended that this checklist be read in conjunction with the SPIRIT 2013 Explanation & Elaboration for important clarification on the items. Amendments to the protocol should be tracked and dated. The SPIRIT checklist is copyrighted by the SPIRIT Group under the Creative Commons "Attribution-NonCommercial-NoDerivs 3.0 Unported" license.

For peer review only

For peer review only - http://bmjopen.bmj.com/site/about/guidelines.xhtml

Appendix B: PARTICIPANT INFORMATION SHEET (PATIENT RECRUITMENT: TRIAL)

Project title: Exploratory randomized trial of face to face and mobile phone counselling against usual care for tobacco cessation in Indian primary care

Principal Investigator and ethics secretary contact number and organization:

Dr Rajmohan Panda, MD, MPH<br>Public Health Foundation of India (PHFI)<br>Additional Professor<br>0124-4781400<br>raj.panda@phfi.org	Dr Aastha Agarwal<br>Member Secretary<br>Research Scientist and Assistant Professor<br>Public Health Foundation of India<br>Email: trc- iec@phfi.org<br>Phone: 0124 478 1400

I am __________________ conducting the research on behalf of Dr. Rajmohan Panda, Additional Professor, PHFI. I am conducting a research to test the effectiveness of face to face and mobile phone counselling against usual care for tobacco cessation in Indian primary care settings. This information sheet describes the research and invites you to be a part of it. You can take time to think about your participation in the research. Before you decide, you can talk to anyone about the research. If this information sheet contains words that you do not understand, please feel free to ask me. If you have any other questions too, you can ask me.

Project brief

Tobacco is an important public health issue contributing towards many significant health problem and needs to be prevented. Tobacco cessation counselling has been identified as an important strategy to help people give up using tobacco. The overburdened health professionals and busy hospital out-patient-department provides limited time to the doctors/counsellors to counsel tobacco users. Through this research we aim to develop and test an innovative mobile telephone counselling system to assist tobacco users give up using tobacco.

What is the treatment offered in this trial?

This research may use mobile phone based counselling system using text messages.

Why have you been asked to take part in this research?

You are being invited to take part in this research because you use tobacco. We request you to consider participating as we feel that you will be able to provide us with information that will help in understanding and improving the mobile counselling services which we will then be tested in a trial. If you agree, I would like to ask you about these matters and request you to answer the questions to the best of your knowledge.

Is my participation in this research entirely voluntary?

Your participation in this research is entirely voluntary. It is your choice whether to participate or not. You do not have to take part in this research if you do not wish to do so, and choosing to participate will not affect your treatment or treatment-related evaluations in any way.

For peer review only

If you do take part, the interview will take about 25 minutes to 40 minutes and I want to assure you that the information that you provide will be kept confidential. Your name or other information that could identify you will not appear in research record or report. You are free to withdraw from this research at any time should you change your mind.

Do you have any questions?

What are the risks of my participation in this research?

We are asking you to share with us some information related to your health and habits, and you may feel uncomfortable talking about some of the topics. You do not have to answer any question you do not want to answer. Nor do you need take part in the intervention or interview if you don't wish to do so, and that is all fine. You do not have to give us any reason for not responding to any question or for refusing to take part in the interview. The information that you give is confidential and will not be shared by any one in any manner that can identify you.

What are the benefits of my participation in this research?

By participating in the research you are contributing in the development of a counselling mechanism which will help people (including yourself) quit tobacco use in the future. This will have direct benefit to your health and that of other people using tobacco.

Will the information I provide be kept private and confidential?

We will ensure your privacy is maintained during the interviews. This will be ensured by conducting one interview at a time in a room in the health facility. We will not share the information you provide with anyone other than the research team. The information so collected from this research will be kept private in a secure manner and none of your personal details will appears on this information. Rather it will be allocated a number which only the researchers will be able to associate the number with you. The information provided by you hence will not be attributed to you by name. The knowledge that we get from this research will be shared with you and your community before it is made widely available to the public.

Do I have the right to refuse or withdraw from the research?

You do not have to take part in this research if you do not wish to do so, and choosing to participate will not affect your treatment or treatment-related evaluations in any way. You may stop participating in the interview at any time that you wish without your treatment being affected.

Respondent agrees for Interview…………………1 ➔ **BEGIN THE INTERVIEW AFTER**

Respondent does not agree for interview…………2 ➔ **END**

For peer review only

CONSENT FORM (PATIENT RECRUITMENT: TRIAL)

Project title: Exploratory randomized trial of face to face and mobile phone counselling against usual care for tobacco cessation in Indian primary care.

Name of Researcher/ Secretary Ethics committee:

Dr Rajmohan Panda, MD, MPH<br>Public Health Foundation of India (PHFI)<br>Additional Professor<br>0124-4781400<br>raj.panda@phfi.org	Dr Aastha Agarwal<br>Member Secretary<br>Research Scientist and Assistant Professor<br>Public Health Foundation of India<br>Email: trc- iec@phfi.org<br>Phone: 0124 478 1400

Please tick the box

1. I confirm that I have read the information sheet for the above research. I have had the opportunity to consider the information, ask questions and have had these answered satisfactorily. ☐

2. I understand that my participation is voluntary and that I am free to withdraw at any time without giving any reason, without my medical care or legal rights being affected. ☐

3. I understand that the information collected about me will be used to support other research in the future, and may be shared anonymously with other researchers. ☐

4. I agree to my primary care practitioner (PCP) being informed of my participation in the research. / I agree to my primary care practitioner being involved in the research, including any necessary exchange of information about me between my PCP and the research team. ☐

5. I agree to take part in the above research. ☐

_______________________________ ___________

Name and Signature (Participant) Date

_______________________________ ___________

Name and Signature (Consent Taker) Date

For peer review only
